# Serum and Urine Metabolic Fingerprints Characterize Renal Cell Carcinoma for Classification, Early Diagnosis, and Prognosis

**DOI:** 10.1002/advs.202401919

**Published:** 2024-07-08

**Authors:** Xiaoyu Xu, Yuzheng Fang, Qirui Wang, Shuanfeng Zhai, Wanshan Liu, Wanwan Liu, Ruimin Wang, Qiuqiong Deng, Juxiang Zhang, Jingli Gu, Yida Huang, Dingyitai Liang, Shouzhi Yang, Yonghui Chen, Jin Zhang, Wei Xue, Junhua Zheng, Yuning Wang, Kun Qian, Wei Zhai

**Affiliations:** ^1^ Department of Urology Renji Hospital School of Medicine in Shanghai Jiao Tong University 160 Pujian Road Shanghai 200127 P. R. China; ^2^ State Key Laboratory of Systems Medicine for Cancer School of Biomedical Engineering and Institute of Medical Robotics Shanghai Jiao Tong University Shanghai 200030 P. R. China; ^3^ Division of Cardiology Renji Hospital School of Medicine in Shanghai Jiao Tong University Shanghai 200127 P. R. China; ^4^ Health Management Center Renji Hospital School of Medicine in Shanghai Jiao Tong University Shanghai 200127 P. R. China

**Keywords:** mass spectrometry, metabolic fingerprinting, prognosis, renal diagnosis, subtype classification

## Abstract

Renal cell carcinoma (RCC) is a substantial pathology of the urinary system with a growing prevalence rate. However, current clinical methods have limitations for managing RCC due to the heterogeneity manifestations of the disease. Metabolic analyses are regarded as a preferred noninvasive approach in clinics, which can substantially benefit the characterization of RCC. This study constructs a nanoparticle‐enhanced laser desorption ionization mass spectrometry (NELDI MS) to analyze metabolic fingerprints of renal tumors (*n* = 456) and healthy controls (*n* = 200). The classification models yielded the areas under curves (AUC) of 0.938 (95% confidence interval (CI), 0.884–0.967) for distinguishing renal tumors from healthy controls, 0.850 for differentiating malignant from benign tumors (95% CI, 0.821–0.915), and 0.925–0.932 for classifying subtypes of RCC (95% CI, 0.821–0.915). For the early stage of RCC subtypes, the averaged diagnostic sensitivity of 90.5% and specificity of 91.3% in the test set is achieved. Metabolic biomarkers are identified as the potential indicator for subtype diagnosis (*p* < 0.05). To validate the prognostic performance, a predictive model for RCC participants and achieve the prediction of disease (*p* = 0.003) is constructed. The study provides a promising prospect for applying metabolic analytical tools for RCC characterization.

## Graphical abstract

The study utilizes nanoparticle‐enhanced laser desorption ionization mass spectrometry to analyze metabolic fingerprints of renal tumors and healthy controls. The metabolic patterns are used to construct multi‐clinical decision models, including classification for tumor detection, malignancy differentiation, precise Renal cell carcinoma (RCC) subtyping, and prognostic evaluation. This work offers a promising outlook for utilizing metabolic analytical tools in characterizing RCC.

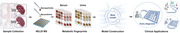

## 1 Introduction

Renal cell carcinoma (RCC) is a major prevalent and deadly type of kidney cancer, with 168 560 new cases and 32 590 deaths in the urinary system reported in the United States.^[^ ^1^ ^]^ Effective management for RCC, including tumor determination, early diagnosis, and prognostic assessment, is vitally important for improving survival outcomes, which helps to enhance the 5‐year survival ratio and guide clinical intervention.^[^ ^2^ ^]^ However, the common characterization methods (e.g., abdominal imaging technologies and liquid biopsy tests) present challenges in small tumor recognition, benign and malignant case distinction, or time‐consuming procedures.^[^ ^3^ ^]^ Meanwhile, the absence of effective biomarkers and prognostic assessment tools hinders the formulation of therapeutic strategies (within 5 years after surgery, ≈20–25% of patients with RCC will encounter the challenges of recurrence and metastasis) and represent low diagnostic specificity.^[^ ^4^ ^]^ Therefore, it is essential to establish a minimally invasive, sensitive, and rapid method for the accurate characterization of RCC.

Liquid biopsy enables real‐time collection of sample information for differential diagnosis, prognostic assessment, and treatment monitoring, which has attracted increasing attention.^[^ ^3^, ^5^ ^]^ Metabolic biomarkers serve as end products of pathways and can potentially offer a more distal characterization of the ongoing pathological process.^[^ ^6^ ^]^ Recently, metabolic biomarkers hold great promise for RCC diagnostic purposes, owing to the least invasive nature of the handling process.^[^ ^7^ ^]^ Nevertheless, current studies based on metabolic profiling mostly focus on single biofluid test, which suffers from the drawback of examining a limited sample cohort, or with a restricted clinical function that neither supports diagnosis nor prognosis.^[^ ^8^ ^]^ Notably, serum metabolomics provides intact metabolic information from the human body, meanwhile, renal tumors occur in the location proximity to the urinary collection. It is foreseen that the integration of serum and urine could enable the identification of potential biomarkers with sufficient clinical relevance.^[^ ^9^ ^]^

Mass spectrometry (MS) is the principal tool that offers accurate molecular information with higher sensitivity and resolution than enzymatic biochemical assays and traditional spectrometric methods. In particular, nanoparticle‐enhanced laser desorption ionization mass spectrometry (NELDI MS) has been widely used in advanced biomedical applications by metabolic fingerprinting of biofluids, such as serum, aqueous humor, cerebrospinal fluid (CSF).^[^ ^10^ ^]^ Compared to liquid/gas chromatography (LC/GC) MS, laser desorption ionization (LDI) MS presents various advantages, including simple sample pretreatment, low sample consumption, high analysis speed, and high throughput.^[^ ^11^ ^]^ Accordingly, NELDI MS platforms provide new opportunities for rapid and accurate metabolic analysis for clinical use in RCC.

**Scheme 1:**
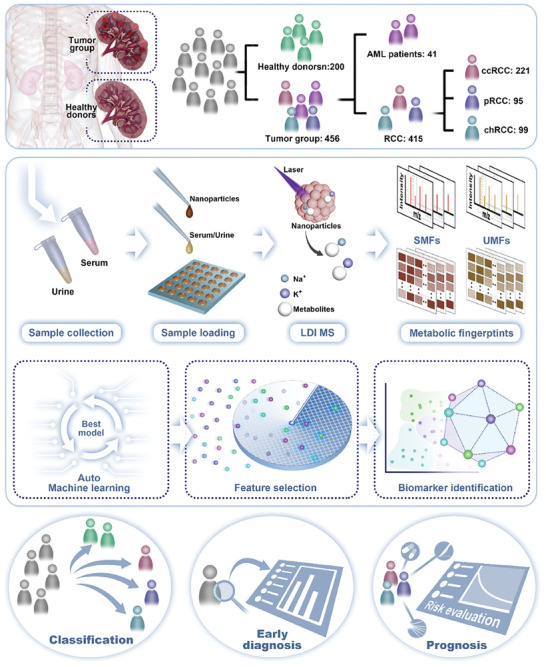
Schematic of biofluid samples characterize RCC for multi‐clinical application by metabolic analysis.

Herein, we acquired metabolic fingerprints of the SUPER (Serum and Urine Programme Established in Renji Hospital) cohort with desirable reproducibility, high throughput, and low sample consumption (**Scheme** **1**). Automated recognition was applied to serum metabolic fingerprints (SMF) and urine metabolic fingerprints (UMF) to distinguish malignant from benign tumors (AML: Angiomyolipoma), classify RCC subtypes (ccRCC: clear cell RCC, pRCC: papillary RCC, chRCC: chromophobe RCC), and achieve the early‐stage diagnosis. Moreover, we identified several metabolic biomarkers for RCC subtype diagnosis, yielding an AUC of 0.813–0.965. Finally, we developed a predictive system to predict the risk of recurrence or metastasis in patients with the major subtype of RCC (ccRCC). Our work has expedited the advancement of metabolic analysis protocols for paired serum and urine, making a valuable contribution to managing RCC in the near future.

## 2 Results

### 2.1 Construction of NELDI‐MS Platform for Metabolic Detection

**Figure 1:**
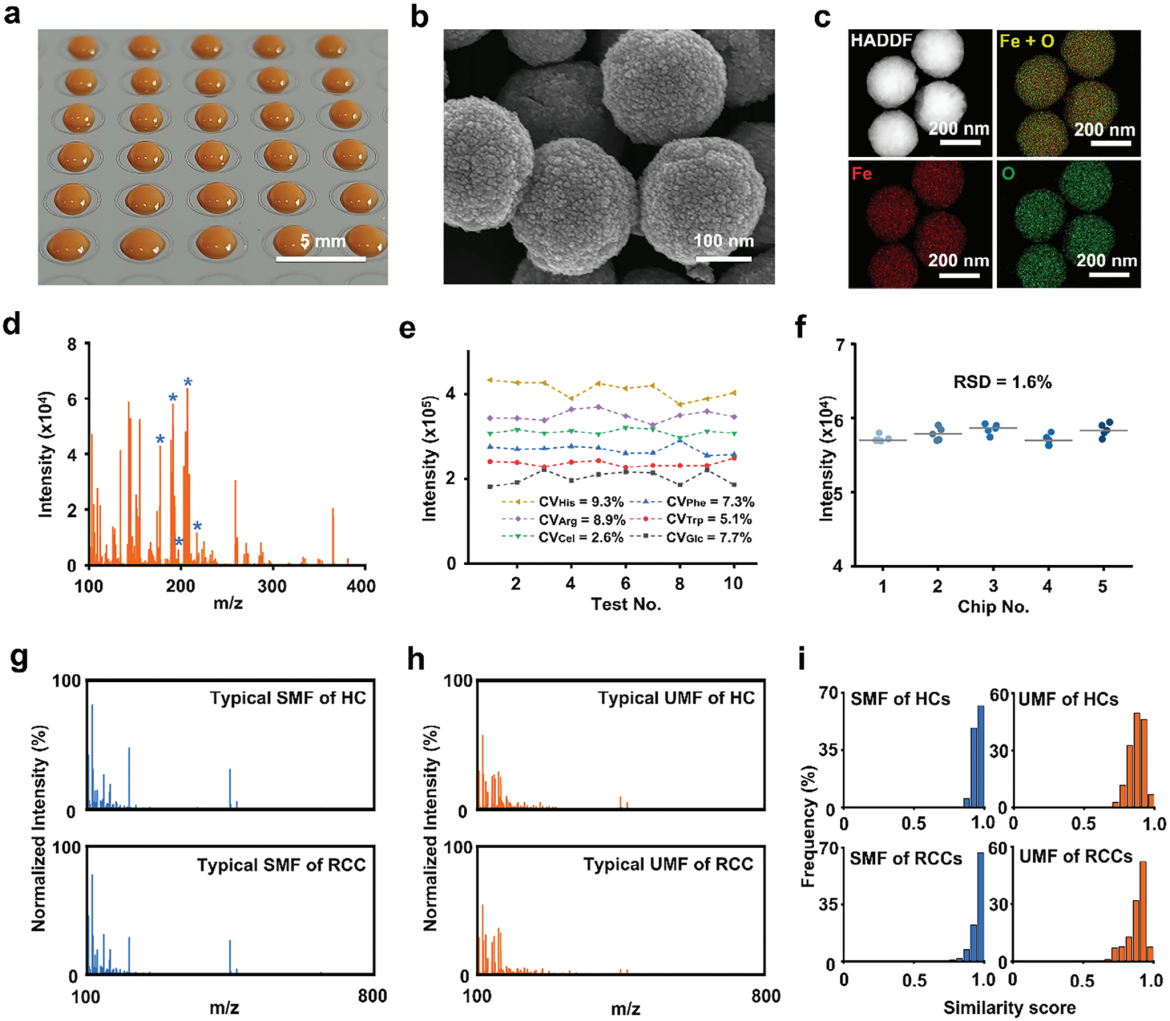
Serum and urine metabolic fingerprints using the NPELDI‐MS platform. a) Digital image of the microarray depicts the printing of the matrix as orange regions on the chip, with a scale bar measuring 5 mm. b) Scanning electron microscopy (SEM) image displays surface cavities of nanoparticles, with a scale bar measuring 100 nm. c) The high‐angle annular dark‐field (HAADF) image and elemental mapping of the nanoparticles show Fe in red and O in green, with the scale bar measuring 200 nm. d) Salt and protein tolerance of the microarray (salt solution: 1 mm PBS; protein: 5 mg mL^−1^ bovine serum albumin (BSA)). Blue asterisks represent histidine (His), phenylalanine (Phe), arginine (Arg), glucose (Glc), and tryptophan (Trp) at the m/z peaks of 178, 188, 197, 203, and 227, respectively. e) CVs of intensities for histidine (His), phenylalanine (Phe), arginine (Arg), tryptophan (Trp), cellobiose (Cel), and glucose (Glc) in a standard mixture over ten tests. f) Microarray reproducibility of five chips for standard Glc (1 mg mL^−1^) intensity in five duplicates. g) Typical mass spectra of the serum metabolic fingerprint (SMF) for HC and RCC in the m/z range of 100–800 Da. h) Typical mass spectra of the urine metabolic fingerprint (UMF) for HC and RCC in the m/z range of 100–800 Da. i) Intragroup similarity scores for serum and urine metabolic fingerprints showed frequency distributions for both HC and RCC patient groups.

We established a ferric oxide‐based NELDI‐MS platform for clinical SMFs and UMFs analysis with rapid analytical speed (≈20 s per sample), low sample consumption (1 µL only), and desirable throughput (384 samples per chip) (**Figure** **1**a; Figure S1a,b, Supporting Information). To construct the high‐performance on‐chip microarray, we prepared ferric oxide nanoparticles as a matrix according to our previous works. Briefly, a low‐cost solution‐thermal method was employed for the synthesis of tailored nanoparticles, and their characterization was conducted using scanning electron microscopy (SEM). The ferric oxide NPs presented polycrystalline and surface roughness structures (Figure 1b), which were suitable as an LDI MS matrix for numerous low‐concentration metabolites analysis. Meanwhile, the HAADF image and elemental mapping of NPs (Figure 1c; Figure S1c, Supporting Information) confirmed its high‐quality polycrystalline nature. To test the NELDI MS platform for the analysis of the mixed metabolites in salt and protein solution, we analyzed five metabolites by LDI MS, including histidine (His), arginine (Arg), phenylalanine (Phe), tryptophan (Trp), and glucose (Glc)).

As shown in Figure 1d, the peaks at m/z of 178, 188, 197, 203, and 227 were assigned to [His+Na]^+^, [Phe+Na]^+^, [Arg+Na]^+^, [Glc+Na]^+^ and [Trp+Na]^+^, respectively. The capability of multiple metabolites analysis and the favorable tolerance to salt and protein using NELDI‐MS was supported by these results, suggesting its potential for direct practical applications without the need for pretreatment. To verify the reproducibility of our platform, we analyzed six metabolites by LDI MS, including His, Arg, Phe, Trp, Glc, and cellobiose (Cel). The coefficient of variations (CVs) of corresponding MS peak intensities at m/z of 178 ([His+Na]^+^), 197 ([Arg+Na]^+^), 365 ([Cel+Na]^+^), 188 ([Phe+Na]^+^), 227 ([Trp+Na]^+^), and 203 ([Glc+Na]^+^), were <10.0% ranging from 2.6% to 9.3% in ten replicates (Figure 1e; Figure S1d–i, Supporting Information). To investigate the reliability of our assay, we conducted a separate study specifically examining the reproducibility and stability of the metabolic fingerprints obtained from the patient sample. We collected ten mass spectra from the same sample at different time points (Figure S7a, Supporting Information), and incorporated intraclass correlation coefficients and comparison of metabolic profiles (Figure S7b, Supporting Information). To further evaluate the reproducibility of the microarray, we detected a standard solution of glucose 1 mg mL^−1^ on five different chips, and the relative standard deviation (RSD) of the MS peak intensity at m/z of 203 ([Glc+Na]^+^) was 1.6% (Figure 1f).

Furthermore, we performed the typical mass spectrum of serum and urine samples from one HC and one RCC patient based on the MS platform, respectively (Figure 1g,h). For each sample, the original mass spectra have ≈57 600 data points from 100–800 Da. Consequently, to demonstrate the similarity in each group, we measured the similarity score of each urine and serum spectra within HC and RCC groups (Figure 1i). Notably, over 90% of samples presented high similarity scores (over 0.8), confirming the detection capability and their spectra similarity in the same group.

### 2.2 Baseline Information and Study Design

**Figure 2:**
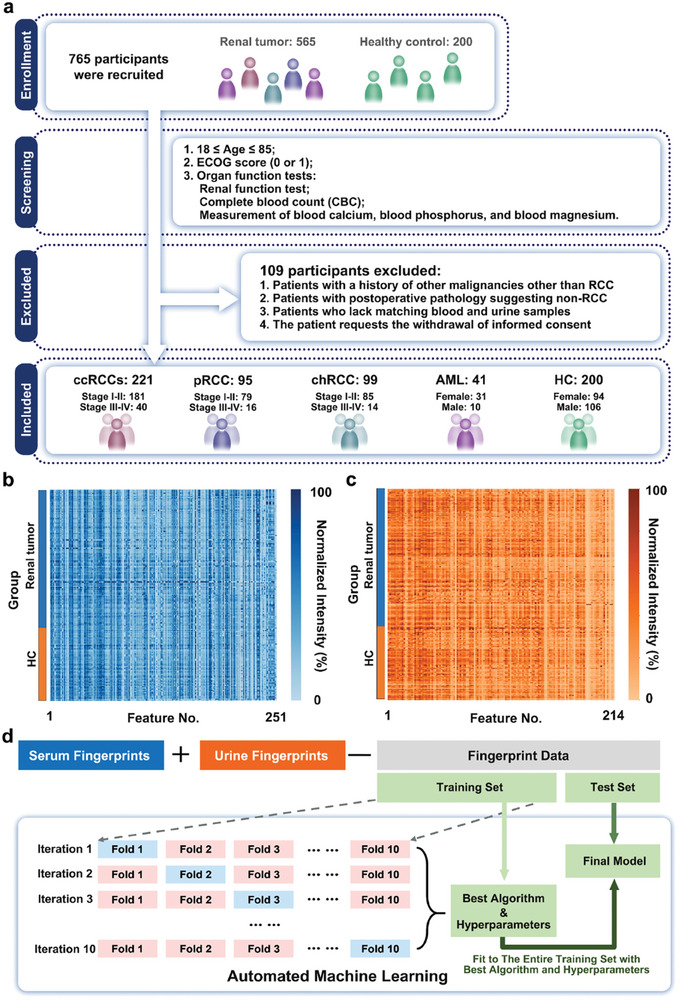
Cohort design and model optimization. a) Workflow summarizing the selection process, where 656 subjects were chosen from 765 subjects. b) SMF of 251 m/z features was extracted from the raw mass spectrometry data. The intensities were displayed using a color scale after logarithmic transformation. c) UMF, including 214 m/z features, was extracted from raw mass spectrometry data. The intensities were displayed using a color scale after logarithmic transformation. d) Workflow of training and evaluation of classification model via automated machine learning.

Within the case‐cohort design, a total of 765 individuals were recruited in this study, comprising 565 subjects from the renal tumor group (Tables S1 and S2, Supporting Information). According to selection criteria (**Figure** **2**a and Supporting Information, Cohort selection), we enrolled 456 renal tumor participants in the study cohort, with 415 malignant tumors as RCC and 41 benign tumors (AML) (**Table** **1**).

**Table 1:** Clinical characteristics of the renal tumor group (*n* = 456).

Clinical indexes		Number	%
Sex			
	Female	210	46
	Male	246	54
Age(years)			
	<60	261	57
	≥60	195	43
BMI			
	<24	176	39
	≥24	280	61
Pathological classification			
	ccRCC	221	48
	pRCC	95	21
	chRCC	99	22
	AML	41	9
Pathological stage (except AML)			
	I	351	85
	II	36	9
	III‐IV	28	6
ISUP grade			
	I	73	23
	II	164	52
	III‐IV	79	25

In the HC group, all participants provided informed consent to participate in this study (Table S3, Supporting Information) without kidney disease, according to the examination results. Then, we collected serum and urine samples from all donors for further analysis using the NELDI MS platform. To construct the SMF and UMF databases, 251 and 214 m/z features were extracted from the original mass spectra. Each database was localized by the highest intensity and peak alignment with the central m/z value and plotted, as shown in Figure 2b,c. For the classification model, we applied an automated machine‐learning method to obtain the best algorithm and optimal hyperparameter combinations. In Figure 1d, we split the united SMF and UMF into training and test sets (7:3) with no significant differences in sex and age (*p* > 0.05), using the nested tenfold cross‐validation technique to identify the optimal algorithm and fine‐tune the hyperparameter combinations in the training set. Subsequently, the final models were selected for application to the test set.

### 2.3 Screening of Tumor Types and RCC Sub‐Categories

**Figure 3:**
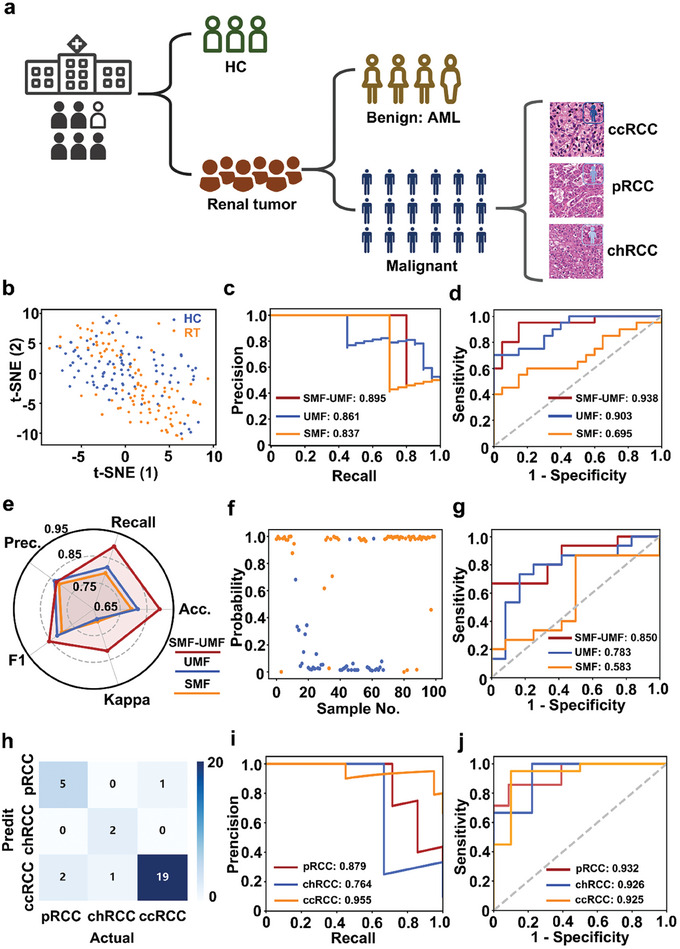
Three‐step diagnostic models. a) Flowchart of the discrimination model for RCC screening. b–d) Screening of Tumor group: b) t‐SNE visualization of the cluster results of the HC (blue) and tumor (orange) groups; c) AUC of the proposed algorithm on the HC and Tumor groups of precision‐recall (PR) curve; and d) receiver operating characteristic (ROC) curve based on three types of biofluid features. e–g) Identification of tumor type: e) Model evaluation of three types of biofluid features based on the final optimized model, including Recall, Accuracy (Acc.), Kappa, F1, and Precision (Prec.); f) a sample‐probability plot for malignant (orange dots) and benign (blue dots); and g) ROC curve for classifying malignant from benign. h–j) Classification of RCC types: h) confusion matrix for the classification results of the proposed algorithm on the validation dataset (7 pRCC, 3 chRCC, and 20 ccRCC); i) PR curve for three subtypes of RCCs (One vs Rest) i,j) ROC curve of three subtypes of RCCs (One vs Rest).

We built a three‐step diagnostic model for tumor group differentiation, benign and malignant tumor identification, and RCC subtype classification using SMF and UMF (**Figure** **3**a).

First, we differentiated tumor groups from healthy controls using unsupervised analysis (t‐distributed stochastic neighbor embedding, t‐SNE). As shown in Figure 3b, the two groups could not be separated, suggesting an improvement in the diagnostic efficiency by machine learning. When the optimal machine learning classifiers were applied in the SMF, UMF, and SMF‐UMF analyses, the SMF‐UMF model showed the desired PR curves with an enhanced AUC of 0.895 (95% CI, 0.854–0.943), compared to the SMF of 0.837 (95% CI, 0.796–0.902) and UMF of 0.861 (95% CI, 0.811–0.928) for the independent validation cohort (Figure 3c). Meanwhile, the ROC results also showed the best performance of the SMF‐UMF model (AUC of 0.938 (95% CI, 0.879–0.967), accuracy of 91.2%, sensitivity of 90.8%, and specificity of 89.9%) in the blind test set, validating the diagnostic value of united SMF and UMF for tumor group screening (Figure 3d).

In the second step, we differentiated malignant tumors from AML. The classification efficiency was evaluated by accuracy, kappa, F1‐score, precision, and recall, which are regarded as vital parameters for the proficiency of the classifier (Figure 3e). The combined SMF and UMF achieved the best performance (accuracy: 0.898, kappa: 0.823, F1‐score: 0.857, precision: 0.815, recall: 0.898). Furthermore, the probability of sample‐level distribution was plotted to demonstrate the excellent discrimination between the malignant tumor (orange) and the benign tumor (blue) using the model ridge classifier in Figure 3f. Additionally, the ROC results in Figure 3g also presented the best classification performance based on the SMF‐UMF, with an AUC of 0.850 (95% CI, 0.824–0.903), accuracy of 84.1%, sensitivity of 83.9%, and specificity of 84.6%) in the test cohort.

In the final step, we achieved RCC subtype discrimination of ccRCC, pRCC, and chRCC using the multi‐classification method after correcting for unbalanced sample sizes (Figure S2, Supporting Information). Figure 3h shows the confusion matrix results of the test set (*n* = 30, ccRCC: 20, pRCC: 7, chRCC: 3). Accurately classified samples (*n* = 26) indicated the multi‐classification efficacy of our model. Importantly, the SMF‐UMF model achieved PR curves with an AUC of 0.879 (95% CI, 0.823–0.918) for pRCC, 0.764 for chRCC (95% CI, 0.733–0.824), and 0.955 for ccRCC (95% CI, 0.932–0.985) (Figure 3i). The model performance was also evaluated via ROC analysis (Figure 3j), achieving an AUC of 0.932 (95% CI, 0.876–0.961) for pRCC, 0.926 (95% CI, 0.874–0.965) for chRCC, and 0.925 (95% CI, 0.882–0.947) for ccRCC.

### 2.4 Classification of Early‐Stage RCC Subtypes

**Figure 4:**
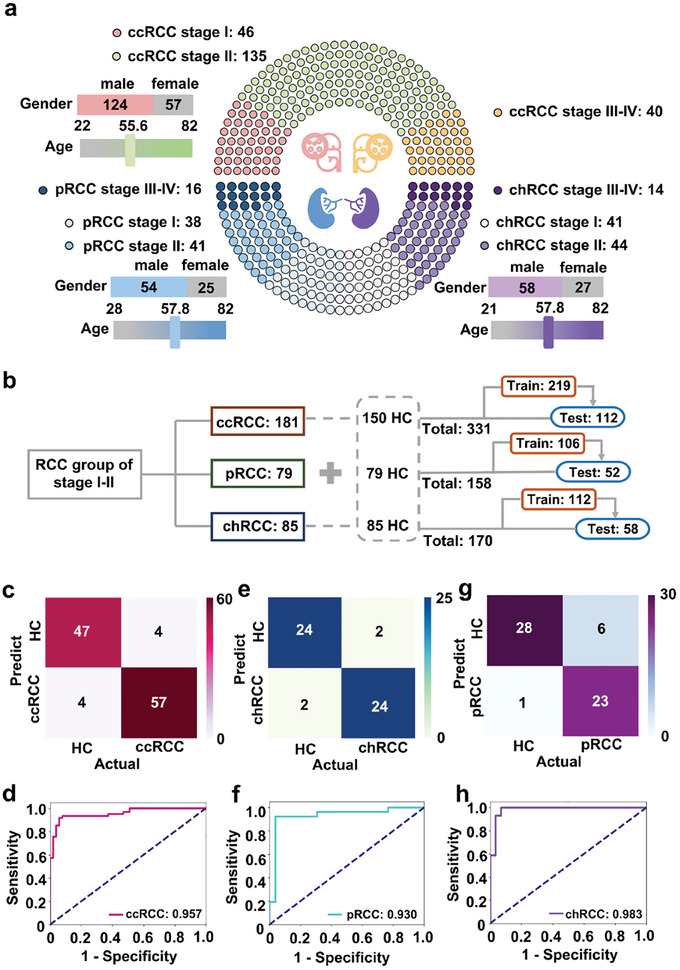
Diagnosis of early‐stage RCC subtypes. a) Age, sex, and stage distribution of 221 ccRCC, 91 pRCC, and 99 chRCC patients. b) Workflow for building diagnostic model. c,d) Confusion matrix for the diagnostic result in the validation group (51 HCs vs 61 early stage ccRCCs) c); Blind test based on the optimized models of Light Gradient Boosting Machine (LGBM) d). e,f) Confusion matrix for the diagnostic result in the validation group (26 HCs vs 26 early stage pRCCs) e) Blind test based on the optimized models of Light Gradient Boosting Machine for validation f). g,h) Confusion matrix for the diagnostic result on the validation group (29 HCs vs 29 early stage chRCCs) g); Blind test based on the optimized models of Ridge Classifier for validation h).

RCC subtype groups containing patients in stages I to IV were divided into ccRCC (*n* = 221), pRCC (*n* = 91), and chRCC (*n* = 99) (**Figure** **4**a; Table S4, Supporting Information). We selected patients with stage I‐II disease and matched HC with no significant difference in age and sex (*p* > 0.05) for model building. Considering the sample size of RCC subtypes, we adjusted the dataset split to 2:1 for training and testing. This strategy ensures sufficient training data for detailed learning while maintaining a substantial test set to assess the model's generalizability, as shown in Figure 4b.

For early‐stage ccRCC diagnosis, 219 samples from the training set can be classified by the LGBM model with optimized hyperparameter combinations, with model performance in Table S5 (Supporting Information). The optimal LGBM model was applied to the test set (*n* = 112). The confusion matrix showed that the model distinguished 57 patients with early‐stage ccRCC from 47 healthy individuals, achieving an AUC of 0.957 (95% CI, 0.913–0.969) with a sensitivity of 92.2% and a specificity of 93.4% (Figure 4c,d).

Regarding the diagnostic performance of early‐stage pRCC, we utilized the training and test sets consisting of 106 and 52 samples, respectively. Similarly, the final model (LGBM) is optimized using the training set (Table S6, Supporting Information). In the test set, the confusion matrix showed a majority of accurate predictions (24/24, Figure 4e). Importantly, we obtained an AUC of 0.930 (95% CI, 0.898–0.956) with a sensitivity and specificity of 0.923 (Figure 4f).

During model building, 170 chRCC patients were divided into training and test sets. The optimal Ridge model (Table S7, Supporting Information) successfully distinguished 23 early‐stage chRCC cases from 28 HC in the test set (*n* = 58) (Figure 4g). Besides, we achieved the performance with an AUC of 0.983 (95% CI, 0.934–0.992), sensitivity of 96.6%, and specificity of 79.3% (Figure 4h, Supporting Information).

### 2.5 Identification of Metabolic Biomarkers for RCC Subtypes

To further explore the promising biomarkers, we filtered the metabolites based on the SMF and UMF databases according to the criteria (mean intensity >1000, model score >0, and *p*‐value <0.05).

**Figure 5:**
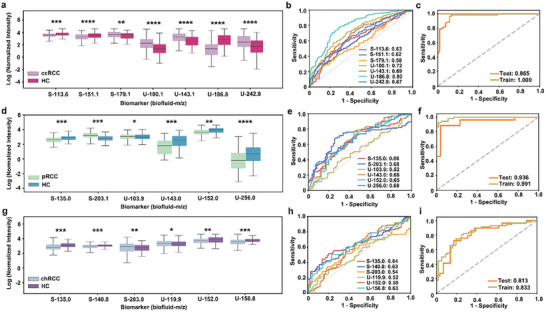
Distribution and diagnostic performance of metabolic biomarkers for three subtypes of RCC. a–c) Biomarker panel for ccRCC: The beeswarm plot demonstrated differential expression of the seven metabolic biomarkers between HCs and ccRCC patients (three biomarkers for serum and four biomarkers for urine). Metabolite levels were presented as normalized intensities after undergoing logarithmic transformation. a); The ROC curves demonstrated the AUCs of each metabolic biomarker (AUC of 0.58–0.80) b); The ROC curves of the metabolic panel for train set and test set to distinguish HC and ccRCC patients c). d–f) Biomarker panel for pRCC: The beeswarm plot demonstrated differential expression of the six metabolic biomarkers between HCs and pRCC patients (two biomarkers for serum and four biomarkers for urine). Metabolite levels were presented as normalized intensities after undergoing logarithmic transformation. d); The ROC curves demonstrated the AUCs of every single metabolic biomarker (AUC of 0.52–0.68) e); The ROC curves of the metabolic panel for train and test sets to distinguish HCs and pRCC patients f). g–i) Biomarker panel for chRCC: The beeswarm plot demonstrated differential expression of the six metabolic biomarkers between HCs and chRCC patients (three features for serum and three features for urine). Metabolite levels were presented as normalized intensities after undergoing logarithmic transformation. g); The ROC curves demonstrated the AUCs of each metabolic biomarker (AUC of 0.52–0.64) h); The ROC curves of the metabolic panel for train set (dark khaki line, AUC = 0.813) and test set (orange line, AUC = 0.833) to distinguish HC and chRCC patients i). ^*^ *p* < 0.05, ^**^ *p* < 0.01, ^***^ *p* < 0.005, and ^****^ *p* < 0.0001.

Specifically, we identified three features from SMF and 4 features from UMF for ccRCC. Compared with HC, the ccRCC group exhibited increased expression levels for S179.1, U100.1, U143.1, and U242.9. In parallel, the ccRCC group demonstrated decreased expression levels of S113.6, S151.1, and U186.8 (**Figure** **5**a). Further, individual variation biomarkers and the combined biomarker panel were applied to evaluate the effectiveness of diagnosis, respectively. The biomarker panel achieved an improved AUC of 0.965 (95% CI, 0.905–0.984) with an accuracy of 94.8%, compared to individual features with a limited AUC ranging from 0.58 to 0.80 (Figure 5b,c).

For pRCC, 2 features were filtered in SMF and 4 features were found in UMF. The expression of S203.1 was elevated, while the others (S135, U103.9, U143, U152, and U256) showed decreased expression levels in the pRCC group (Figure 5d). Moreover, the classification model demonstrated improved overall performance in distinguishing pRCC from the HC group, with an AUC of 0.936 (95% CI, 0.879–0.974) and accuracy of 91.9%), the result surpassed the analysis of a single metabolic feature, where AUC ranged from 0.52 to 0.68) (Figure 5e,f).

Furthermore, we analyzed six feature levels between the chRCC and HC. Among them, S203 and U119.9 were up‐regulated, while S135, S140.8, U152, and U156.8, were down‐regulated in chRCC compartments compared with HC (Figure 5g). The aforementioned features demonstrated an AUC range of 0.52–0.64 in their ability to distinguish chRCC from HC. The combined panel results in a significantly improved AUC of 0.813 (accuracy of 79.8%) for the validation cohort (Figure 5h,i).

Notably, we investigated potential metabolic pathways associated with serum and urine metabolites (Tables S9 and S10, Supporting Information). There were ten relevant pathways with pathway impact (PI) > 0 and hit number (the number of metabolites matched to each pathway) ≥1, including: 1) Warburg Effect, 2) Sphingolipid Metabolism, 3) Galactose Metabolism, 4) Gluconeogenesis, 5) Glycolysis, 6) Transfer of Acetyl Groups into Mitochondria, 7) Lactose Synthesis, 8) Glucose‐Alanine Cycle, 9) Trehalose Degradation, and 10) Lactose Degradation (Figure S7, Supporting Information). The above pathway analysis suggested that the screened metabolites play a significant role in regulating glucose levels and amino acid metabolism.^[^ ^12^ ^]^

### 2.6 Performance of Biomarker Panel for CCRCC Diagnosis

**Figure 6:**
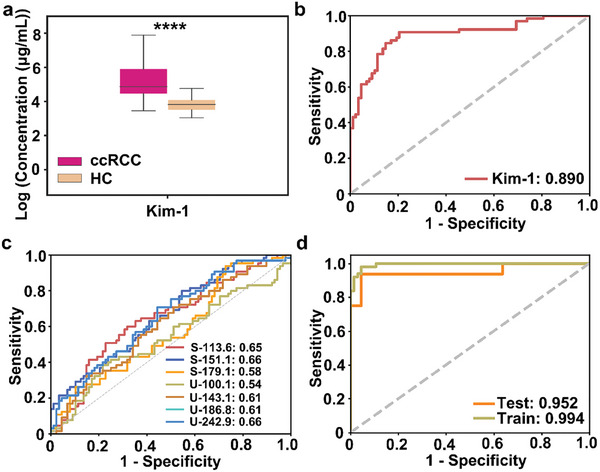
Diagnostic potential for ccRCC patients. a) Plasma levels of KIM‐1 for HC and ccRCC; b) The ROC curve of KIM‐1 for discriminating between HC and ccRCC. c) The ROC curves showed the performance of each metabolic biomarker; d) The ROC curves showed the performance of each metabolic biomarker.

To confirm the clinical value of SMF‐UMF for ccRCC diagnosis, we selected a verification cohort with ccRCC (*n* = 65) and HC (*n* = 88) with no significant differences in sex and age (*p* > 0.05). Kidney Injury Molecule‐1 (KIM‐1) is a potential clinical indicator for kidney function and was selected for comparison. In **Figure** **6**a, KIM‐1 was overexpressed in the plasma of ccRCC patients compared to HC, with a diagnostic AUC of 0.89 (Figure 6b). In contrast, the biomarker combination of ccRCC achieved discrimination with an enhanced AUC of 0.952 (95% CI, 0.896–0.989) for the test set and 0.994 for the discovery cohort, respectively, compared to individual features with the limited AUC of 0.54–0.66 (95% CI, 0.501–0.702) (Figure 6c,d).

### 2.7 Prognostic Value of Serum and Urine Metabolic Fingerprints

**Figure 7:**
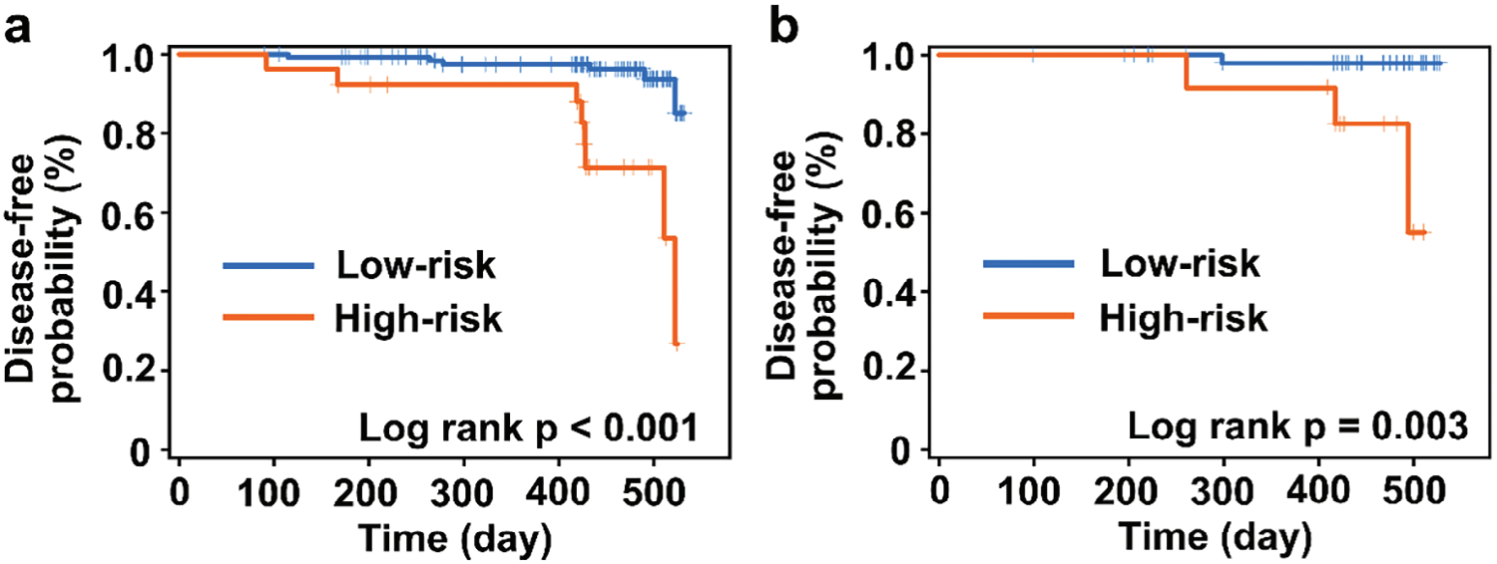
Disease‐free analysis for ccRCC of low‐risk and high‐risk groups. a) training set; b) test set.

Specifically, we assessed the predictive ability of the diagnostic model using the Kaplan–Meier curve in the ccRCC group (*n* = 221). As a result, the low‐risk group showed a significantly prolonged median disease‐free time compared to the high‐risk group, as indicated by the log‐rank test results (*p* < 0.001 in the training set, **Figure** **7**a; *p* = 0.003 in the test set, Figure 7b; *p* < 0.001 according to the TNM staging system, Figure S5, Supporting Information), demonstrating the favorable prognostic performance of SMF‐UMF.

## 3 Discussion

Differential diagnosis, early detection, and prognostic assessment for RCC emerge as crucial global concerns. Recently, several types of body fluid markers have played a prominent role in the diagnosis and prognosis of renal diseases, ranging from genomic, and proteomic, to metabolomic levels. The upstream biomarkers of nucleic acids and proteins are used for the management of RCC and showed AUCs of 0.70–0.85^[^ ^13^ ^]^ with biochemical reaction‐based signal amplification assays. Additionally, elevated levels of carbonic anhydrase IX (CA‐IX) expression have been consistently observed in tissue samples of RCC patients with constitutive activation of hypoxia‐inducible factor (HIF).^[^ ^14^ ^]^ In our study, a typical examination of a ccRCC patient's tumor tissue (Figure S9, Supporting Information) revealed a positive expression of CA‐IX. This suggests the likelihood of an aggressive tumor and potentially a poorer prognosis. Such findings could be utilized in conjunction with biofluid markers as a complementary approach to enhance the accuracy of RCC diagnosis. Downstream products of metabolic biomarkers are expected to provide real‐time insight into phenotypic variations in biological systems, making them highly accessible targets for clinical intervention. Of note, previous studies have demonstrated promising results for employing metabolic biomarkers for diagnosing RCC but remain inconclusive.^[^ ^15^ ^]^ Notably, work design plays a fundamental role in providing precise insights into disease phenotype by selecting proper bio‐fluids and enrolling an appropriate cohort.

For biofluid selection, urine can be collected less invasively than other biofluids and could serve as a highly appropriate biological resource for large‐scale clinical studies, which has been applied to explore RCC status and metabolic pathways for RCC diagnostic analyses. Current studies suggest that blood circulation and urine collection correlate with perturbations in glycolysis, tricarboxylic acid (TCA) cycle, amino acid metabolism, and fatty acid metabolism.^[^ ^16^ ^]^ Accordingly, paired serum and urine were enrolled in this study, our results demonstrate that the best performance for managing RCC was achieved by combining these two biofluids. To our knowledge, few epidemiological studies have validated the assumption by comparing single biofluids (serum or urine) and combined biofluids for their utility using metabolomics.

For cohort design, previous studies on metabolic analysis of RCC have mainly utilized a limited cohort size, with a sample number of 50–400 for investigation.^[^ ^15^, ^17^ ^]^ In comparison, our study enrolled a total of 656 individuals with clear collection criteria. Subsequently, using a large‐scale data mining approach (auto‐learning algorithm) for feature selection and model development, we attained an enhanced AUC of 0.898 for tumor type classification with SMF‐UMF compared to individual metabolic fingerprints (SMF of 0.585, UMF of 0.783). Encouraged by the improved performance of unified metabolic fingerprints, we also applied them in the subtype classification and early‐stage diagnosis of RCC. Further, we identified several metabolic features to construct a specific panel for RCC subtype diagnosis. These results strongly indicate that our metabolic platform can serve for RCC management and is valuable for extensive clinical applications, owing to the appropriate choice of biofluids, advanced machine learning methods, and a well‐considered cohort design.

Finally, developing an efficient prognostic model to accurately identify high‐risk patients is essential for guiding therapeutic decision‐making. Liquid biomarkers, such as circulating tumor cells (CTCs), microRNAs (miRNAs), and cf‐DNA/ct‐DNA, correlate with overall survival (OS) and progression‐free survival (PFS), enabling the prediction of patient survival. These biomarkers have the potential to assist in identifying high‐risk patients and predicting the risk of metastasis and recurrence, with a *p*‐value less than 0.005.^[^ ^18^ ^]^ In contrast, a prognostic model was constructed in this study by implementing Cox regression analysis of combined SMF‐UMF data, demonstrating a comparable prognostic efficiency with a *p*‐value of 0.003. Therefore, the SMF‐UMF prediction model only requires NELDI MS analysis, indicating its potential as a real‐time detection tool for large‐scale applications and its ability to improve disease management.

Nevertheless, there are some limitations to consider in this work. First, this study was conducted at a single center. Further research is needed to include larger sample sizes from multiple centers, covering diverse geographic regions and racial backgrounds. Second, the biological significance of metabolites remains unclear and requires comprehensive clarification. A deep understanding of the relationship between metabolites and the development of renal tumors may indirectly uncover potential targets for metabolic interventions in clinical therapy. Third, the integration of advanced instrumental techniques has the potential to improve the specificity and confidence in detecting metabolic analysis. For instance, designer nanoparticles play a crucial role in NELDI MS and can be tailored for specific practical applications. These approaches could potentially offer opportunities to screen a broad population with renal tumors, provide therapies at a more treatable phase, and thus alleviate the physical and emotional burden on patients while reducing the economic burden on society.

## 4 Experimental Section

### Sample Harvesting

This work was approved by the Ethics Committee of Shanghai Renji Hospital (Shanghai, China, IRB KY2022‐001‐B) and was conducted by the Declaration of Helsinki. All the paired biofluid samples were obtained with written informed consent. For sample collection, 5 mL peripheral venous blood samples and 15 mL urine samples per person were obtained from all the patients with renal tumors and healthy donors. All the collected samples were transferred to the laboratory for additional processing within an hour at 4 °C. The blood samples were centrifuged at 3000 rpm at 4 °C for 10 min, and the urine samples were centrifuged at 3000 g at 4 °C for 10 min. Following centrifugation, the plasma from the upper layer of the blood sample and the supernatant solution from the urine sample were collected and stored at −80 °C. Each tube of the sample was carefully labeled to ensure that the specimen was consistent with the patient.

### MS Experiments

The ferric nanoparticles were used as the matrix for NELDI MS. They were prepared in deionized water, reaching a concentration of 1.0 mg mL^−1^. Before the mass spectrometry (MS) analysis (refer to Supporting Information for the construction of the NELDI‐MS platform for metabolic detection), each biofluid sample (serum or urine) required ≈15 s for careful dilution. Next, a minute volume of the analyte solution and an equivalent quantity of the matrix slurry (1 µL per spot) was dispensed onto the chip, followed by drying under ambient conditions. This drying process took ≈90 min for each batch of 384 samples (equivalent to 14 s per sample). The mass measurements were calibrated using standard small molecules, and positive ion mode was employed for all MS experiments. The instrumental parameters were optimized to ensure optimal performance. Each analysis involved a laser pulse frequency of 1 kHz, 2000 laser shots, a delay time of 200 ns, and an acceleration voltage of 20 kV. To identify crucial biomarkers, the HPLC‐MS instrument was used for MS analyses, while the TOF‐MS system was employed for MS/MS experiments.

### MS Data

Preprocessing of the raw mass spectra data was conducted using a custom‐built code before applying machine learning techniques in Python (version 3.8). The preprocessing steps included peak detection, peak alignment, peak filtration, standardization, and peak filtration. The discrete wavelet transform was applied for spectrum smoothing and baseline correction. The local maximum was used for peak detection, and signal‐to‐noise ratio, intensity, and shape ratio (peak area) were employed for peak filtration. No standardization was used in the data preprocessing process. No missing value imputation or internal standards were used. The united database was constructed using the concatenation method. the SMF and UMF were aligned and combined along the sample axis, effectively expanding the informational breadth without changing the sample size. For diagnosis, machine learning was applied to analyze the metabolic fingerprints of serum and urine, incorporating feature selection and model construction. Feature selection was applied to eliminate redundant collinear mass‐to‐charge ratio (m/z) signals by identifying the most predictive variables using an elastic net algorithm, given the high dimensionality of the metabolic fingerprints (SMF and UMF). The threshold of the coefficient was optimized for each machine‐learning algorithm during the feature selection process. The linear regression algorithm incorporated L1 and L2 regularization on the coefficients as a penalty term in the loss function. This adjustment helped drive the coefficients of weak m/z signals closer to zero. the performance of Lasso, Ridge, and ElasticNet was evaluated on each database by utilizing a held‐out test set during the feature selection process, which was done by computing the lowest Mean Squared Error (MSE) value. To automate the intricate and time‐consuming clinical tasks, Automated Machine Learning (AutoML) was utilized for model selection and hyperparameter tuning using PyCaret. This platform can quickly train a set of predefined models on preprocessed data. It then selects an appropriate search strategy, such as grid search, random search, or more advanced techniques like Bayesian optimization, to fine‐tune model parameters. This methodology involved the orchestration of various machine learning algorithms, including Light Gradient Boosting Machine (LGBM), Extra Trees (ET), Random Forest (RF), Ada Boost (AB), Gradient Boosting (GB), Decision Tree (DT), K Neighbors (KNN), Logistic Regression (LR), Linear Discriminant Analysis (LDA), and Naive Bayes (NB). For validation, the best machine learning algorithm was evaluated in the independent validation cohort to study the overfitting effect. AutoML helps determine the best model for each clinical task, as well as optimal parameter combinations for each algorithm (summarized in Table S5, Supporting Information).

### Statistical Analysis

The t‐distributed stochastic neighbor embedding (t‐SNE), precision‐recall (PR) curve, receiver operating characteristic (ROC) curve, confusion matrix of classification, cosine similarity, and correlation analysis were performed using Python version 3.8. Metabolic analysis was performed using MetaboAnalyst 5.0 (https://www.metaboanalyst.ca/), and the verification of the metabolites showing a significant difference among RCC patients was conducted on the Human Metabolome Database (HMDB, http://www.hmdb.ca/) based on the accurate molecular formula. The similarity score was calculated using the adjusted cosine similarity algorithm. In this study, additional statistical analyses were conducted using the SPSS software (version 24.0, SPSS Inc., USA) to calculate the statistical significance (*p*‐value). These analyses included the two‐sided Student's *t*‐test, chi‐square test, and ANOVA. A significance level of 0.05 was established. For the prognosis, a multivariate Cox regression analysis was conducted on SPSS software, version 22.0, in SMFs‐UMFs of the discovery cohort. Further analysis only considered markers with a *p*‐value <0.05. Afterward, a linear regression predictor was generated using the selected biomarkers. Kaplan–Meier survival analysis was conducted to evaluate the prognostic effectiveness of the TNM stage using R (version 3.4.3) with the “survival” and “timeROC” packages. The Human Metabolome Database (HMDB) and PubChem were used to search for candidate metabolites for biomarker identification. In this work, additional statistical analyses were conducted using the SPSS software to calculate the *p*‐value for statistical significance. The following tests were employed: the two‐sided Student's *t*‐test, the Mann–Whitney U test, and the paired *t*‐test. These tests were used for statistical analysis to ensure the significance of the results. The significance level was set at 0.05.

## Conflict of Interest

The authors declare no conflict of interest.

## Author Contributions

X.X., Y.F., and Q.W. contributed equally to this article. Z.J., W.Y., Q.K., and Z.W. conceptualized and obtained funding for the study. F.Y., D.Q., and G.J. collected clinical samples. W.Q., Z.S., L.W., C.Y., Z.J., and X.W. conducted clinical research. X.X. analyzed clinical data and drafted the manuscript. L.W., W.R., Z.J., H.Y., L.D., and Y.S. were involved in data analysis and material characterization. All authors contributed to revising the manuscript and approved the final version as submitted.

## Supporting information

Supporting Information

## Data Availability

The data that support the findings of this study are available from the corresponding author upon reasonable request.
