# Supplementary material for: Serum and Urine Metabolic Fingerprints Characterize Renal Cell Carcinoma for Classification, Early Diagnosis, and Prognosis
Source: Adv Sci (Weinh). 2024 Jul 8;11(34):2401919. doi: 10.1002/advs.202401919 (PMC11425863; doi:10.1002/advs.202401919)
Supplement: Supplementary file 1 — Supporting Information [file ADVS-11-2401919-s001.docx]

**Supporting Information (SI)**

Serum and urine metabolic fingerprints characterize renal cell carcinoma for classification, early diagnosis, and prognosis

Xiaoyu Xu^b,c†^, Yuzheng Fang^a†^, Qirui Wang^d†^, Shuanfeng Zhai^a^, Wanshan Liu^b,c^, Wanwan Liu^d^, Ruiming Wang^b,c^, Qiuqiong Deng^d^, Juxiang Zhang^b,c^, Jingli Gu^d^, Yida Huang^b,c^, Dingyitai Liang^b,c^, Shouzhi Yang^b,c^, Yonghui Chen^a^, Jin Zhang^a^, Wei Xue^a^, Junhua Zheng^a*^, Yuning Wang^b,c*^, Kun Qian^b,c*^, Wei Zhai^a*^

^a^Department of Urology, Renji Hospital, School of Medicine in Shanghai Jiao Tong University; 160 Pujian Road, Shanghai 200127, China

^b^State Key Laboratory for Oncogenes and Related Genes, School of Biomedical Engineering and Institute of Medical Robotics, Shanghai Jiao Tong University, Shanghai 200030, China

^c^Division of Cardiology, Renji Hospital, School of Medicine of Shanghai Jiao Tong University, Shanghai 200127, China

^d^Health Management Center, Renji Hospital, School of Medicine in Shanghai Jiao Tong University, Shanghai 200127, China

This section includes the following:

Methods

Figure S1-S9

Table S1-S10

**Methods**

**Chemicals and reagents**

The constituents employed in this study can be classified into three distinct categories: agents employed for nanoparticle synthesis, standard metabolites utilized for detection, and miscellaneous salts and proteins. For nanoparticle synthesis, the ferric chloride hexahydrate (97%), trisodium citrate dihydrate (99%), anhydrous sodium acetate (99%), and ethylene glycol (99.5%) were procured from Sinopharm Chemical Reagent Beijing Co., Ltd. (Beijing, China). For standard metabolites, Histamine (His, 97%), D-(+)-cellobiose (Cel, 98%), D-glucose (Glu, 99.5%) were ordered from Sigma-Aldrich (St. Louis, MO, USA). Phenylalanine (Phe, 98%) was acquired from Inno-chem Co., Ltd (Beijing, China). L-arginine (Arg, 99%) and L-Tryptophan (Trp, 98%) were purchased from Aladdin Reagent (Shanghai, China). For salts and protein, sodium chloride (NaCl, 99.5%) and potassium chloride (KCl, 99.5%) were ordered from Sigma-Aldrich (St. Louis, MO, USA), and bovine serum albumin (BSA, 95%) was purchased from Beijing Solarbio Science & Technology Co., Ltd. (Beijing, China). All chemical substances and reagents utilized in this study were employed in their pristine form, devoid of any subsequent purification or enrichment procedures. For all experimental aqueous solutions, deionized water (18.2 MΩ·cm, Milli-Q, Millipore, GmbH) was scrupulously employed for preparation.

**Nanoparticle preparation and characterization**

For preparation, a refined solvothermal technique was implemented to facilitate the extensive-scale fabrication of ferric nanoparticles (1, 2). Initially, the ferric chloride hexahydrate was dissolved within an ethylene glycol solution, followed by the addition of trisodium citrate dihydrate and anhydrous sodium acetate to the same solution. Subsequently, the solution underwent sonication at ambient temperature for 45 minutes. Thereafter, the resulting solution was carefully transferred to a Teflon-lined stainless-steel autoclave, where it underwent a hydrothermal reaction under elevated temperatures of 200 °C for 10 hours. Upon cooling down to room temperature, the final product was collected and subjected to sequential washes with ethanol and deionized water before being dried for 24 hours at 60 °C, rendering it suitable for subsequent applications. For characterization, the nanoparticle suspension was first printed on aluminum foil, after which the scanning electron microscopy (SEM) images were acquired using the S-4800 (Hitachi Ltd., Japan). For transmission electron microscopy (TEM) and elemental mapping analysis, the cutting-edge JEOL 2100F (JEOL Ltd., Japan). The nanoparticle suspension was carefully deposited onto a copper grid with a mesh size of 200. The advanced UV 3600 (Shimadzu Ltd., Japan) helps to acquire the optical spectrum after the nanoparticles are deposited onto the glass slide. Finally, the digital image of the mass spectrum chip was captured using a Xiaomi 12S smartphone (Xiaomi Technologies Co., Ltd., China).

**MS standard**

The comprehensive analysis of standard metabolites, serum, and urine samples were conducted on the Bruker systems with Nd: YAG lasers (355 nm) for Autoflex (Time of Flight-Mass Spectrometry, TOF-MS) and Solarix 7.0T (Fourier Transform-Ion Cyclotron Resonance-Mass Spectrometry, FT-ICR-MS), using prepared nanoparticles for LDI MS process.

**Cohort selection**

The inclusion criteria for participants encompassed the following aspects:

1. Subjects were required to provide voluntary participation in the study and present signed and dated written informed consent before engaging in study-specific procedures, sampling, and analysis. They were also expected to adhere to compliance regulations and demonstrate cooperation during follow-up.

2. Participants falling within the age range of 18 to 85 years were considered eligible for inclusion.

3. An Eastern Cooperative Oncology Group (ECOG) score of 0 or 1 was a prerequisite for participation, indicating well-maintained overall performance status.

4. Eligible participants needed to exhibit normal functioning of major organs, as determined by meeting the below criteria: (i) White blood cell count (WBC) ≥ 4*10^9/L; neutrophil count ≥ 2*10^9/L; hemoglobin (HB) level ≥ 90 g/L; platelet count (PLT) ≥ 100*10^9/L. (ii) No evidence of functional organic diseases, as indicated by the fulfillment of the following criteria: total bilirubin (T-BIL) ≤ 1.5 times the upper limit of normal (ULN); alanine aminotransferase (ALT) and aspartate aminotransferase (AST) levels ≤ 2.5 times the ULN; creatinine levels ≤ 2 times the ULN, with an endogenous creatinine clearance greater than 30 ml/min (calculated using the Cockcroft-Gault equation); international normalized ratio (INR) and activated partial thromboplastin time (aPTT) ≤ 1.5 times the ULN. (Note: ULN denotes the upper limit of normal.)

In addition to the inclusion criteria mentioned above, we use certain exclusion criteria to ensure the reliability and validity of study findings. The exclusion criteria were as follows:

1. Patients with any other concomitant malignant tumors besides renal cell carcinoma were excluded from the study.
2. Patients who had received any form of antitumor therapy within 4 weeks before enrollment was excluded to avoid the potential confounding effects of these treatments on the metabolic profiles.
3. Patients with active infections, including systemic or localized infections, were excluded as these could impact the metabolic fingerprints and introduce variability in the results.
4. Patients with a history of kidney transplantation or any other malignancies were excluded to maintain the homogeneity of the study population.

**Feature selection**

1. Feature and Label Split: This function splits the DataFrame into a feature matrix X (containing all feature columns) and a target vector y (representing the label column).
2. Data Standardization: To ensure all features have the same scale and to improve model performance, the code applies StandardScaler from sklearn.preprocessing. Each feature has a mean of 0 and a standard deviation of 1.
3. Train-Test Split: The standardized data (features normalized from 0 to 1) is divided into training and testing sets. A 70%-30% split is performed, with 30% of the data reserved for testing. Random state 42 is set for reproducibility.
4. Regularization Parameter Grid: A grid of regularization strengths (alphas) is created ranging from 10^-4^ to 10^4^ in 50 logarithmically spaced steps. These values will be explored during model tuning to find the optimal regularization strength.
5. Model Definition and Hyperparameter Tuning Setup: Three linear models are defined along with their hyperparameter grids (Lasso: alpha; Ridge: alpha; ElasticNet: Incorporates both alpha and l1_ratio, the latter determining the mix between L1 and L2 penalties). The grid specifies three different l1_ratio values.
6. Cross-Validation and Model Evaluation: GridSearchCV is used to conduct 5-fold cross-validation for each model. It tunes the models based on the negative mean squared error, aiming to minimize this cost function. The best model configuration (with the lowest cross-validated Mean Squared Error) is selected for each algorithm.

**Comparison of regularization methods**

Mean Squared Error (MSE) was used to compare the effectiveness of regularization methods (Lasso, Ridge, and ElasticNet). Figure S3 illustrates that ElasticNet achieved the lowest MSE, which can be attributed to the fact that L1 regularization might oversimplify and overlook some weak yet valuable features. In contrast, L2 regularization typically evens out the weights of all features, thereby reducing the model's complexity without entirely discarding any features. By simultaneously incorporating L1 and L2 penalty terms, ElasticNet can enhance generalization ability and ensure a more balanced distribution of feature weights. This approach maintains feature sparsity while preserving information from weaker features, leading to improved generalization and a more reasonable allocation of feature weights. Therefore, we utilize the ElasticNet algorithm to balance the L1 and L2 penalty terms. By tuning the "l1_ratio" parameter, we aim to identify the most informative feature subset while mitigating overfitting and managing multicollinearity.

**Strategies for mitigating overfitting**

1. Cross-validation: 10-fold cross-validation techniques during the model training and hyperparameter tuning phases.

2. Regularization techniques: Elastic Net introduces a penalty term to the model's objective function, discouraging large weights and thereby preventing the model from excessively fitting noise in the training data.

3. Early stopping criteria: By monitoring the performance on a validation set and halting training once improvements plateaued or began to degrade, we curtailed unnecessary fitting to the training data.

4. Feature selection: removing redundant or irrelevant features that could contribute to overfitting.

5. Comprehensive performance evaluation: multiple performance metrics, including not just accuracy but also precision, recall, F1-score, and area under the ROC curve, and all the values calculated by the DeLong test were less than 0.05.

**Multi-classification analysis**

1. Data resampling: We initiated the process by implementing the Synthetic Minority Over-sampling Technique (SMOTE) to address class imbalance issues in our dataset. This technique generates synthetic samples for the minority class to balance it with the majority class, enhancing the model's learning capacity on underrepresented data. Through rigorous experimentation, we determined that setting "k_neighbors = 2" for SMOTE yielded the optimal balance between synthetic data diversity and fidelity, as evidenced by performance metrics depicted in Figure S2.
2. Stratified Sampling Strategy: To ensure that the class distribution was maintained across all subsets of data used during model validation and testing, we employed a stratified sampling approach. This meant that each fold in the cross-validation process, as well as the final test set, contained a proportionate representation of all classes similar to the original dataset. By doing so, we guaranteed that our model's evaluation was not biased by imbalanced data subsets and provided a more accurate assessment of its ability to generalize across all classes. This strategy is particularly crucial in multi-classification scenarios where maintaining class balance is vital for understanding true model performance.

**Figure S1**


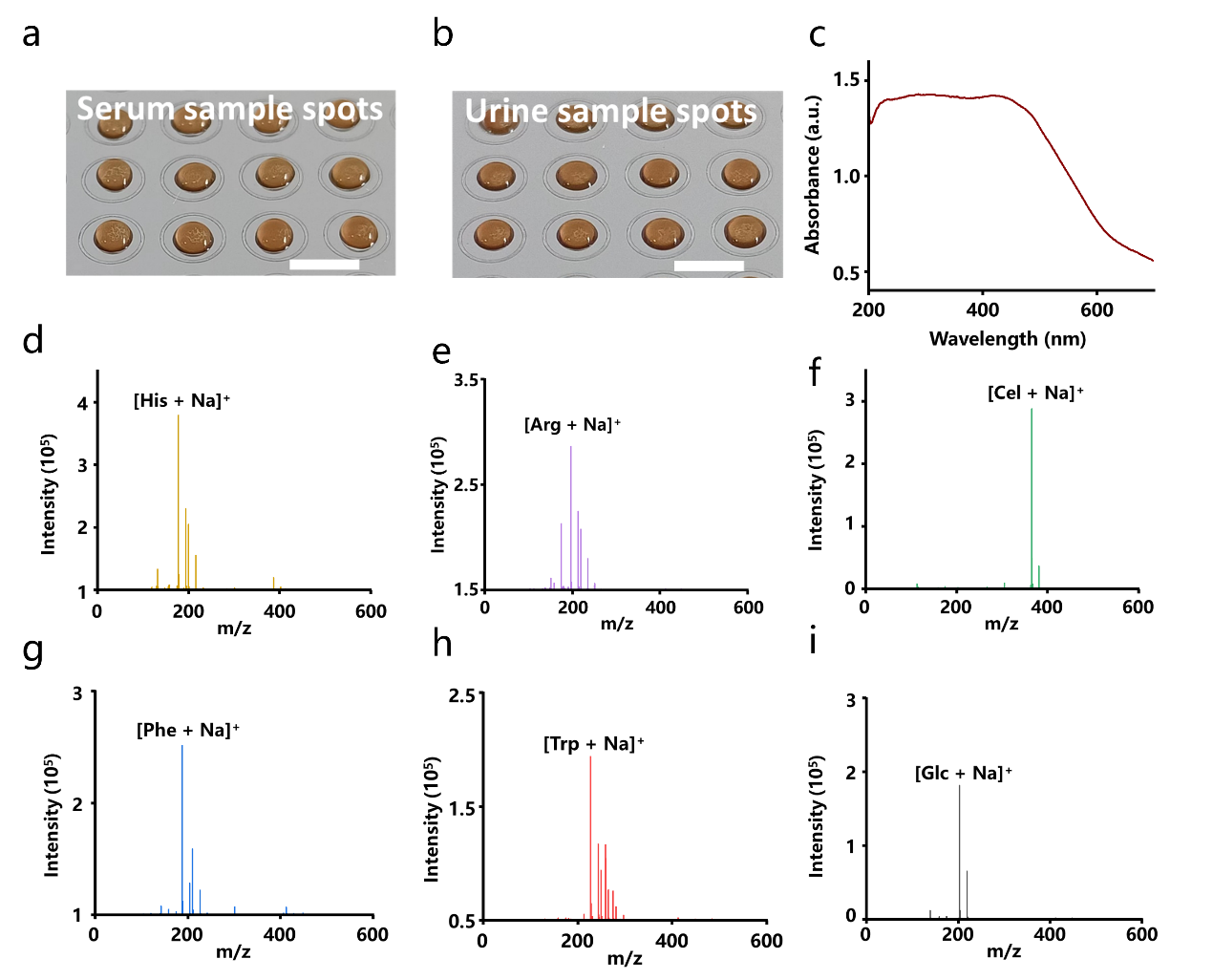


**Figure S1**

Characterization of microarray and the ferric nanoparticles. (a-b) Digital images of MS microarrays showed the MS chips after serum (a) and urine (b) sample printing, respectively. All scale bars were 5 mm. (c) Optical extinction spectrum of the NPs from 200 to 700 nm. (d-i) Typical MS of metabolites samples: 1 mg/mL of His (c), Arg (d), Cel (e), Phe (f), Trp (g), and Glc (h), respectively.

**Figure S2**


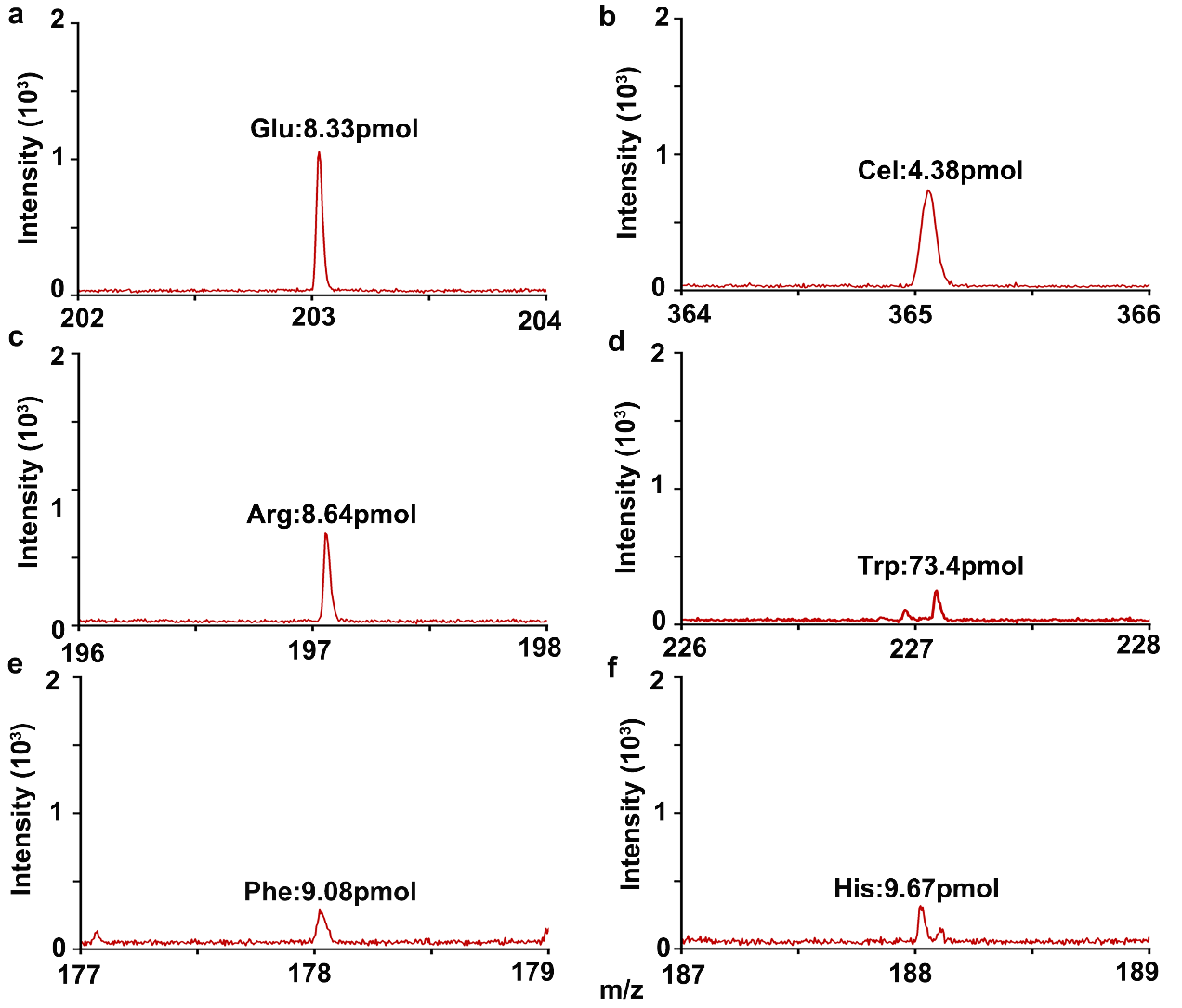


**Figure S2**

The detection limit of specific metabolites. Typical mass spectra of a) Glc (0.001 mg/mL, 1.5 μL), b) Cel (0.001 mg/mL, 1.5 μL), c) Arg (0.001 mg/mL, 1.5 μL), d) Trp (0.01 mg/mL, 1.5 μL), e) Phe (0.001 mg/mL, 1.5 μL), and f) His (0.001 mg/mL, 1.5 μL).

**Figure S3**


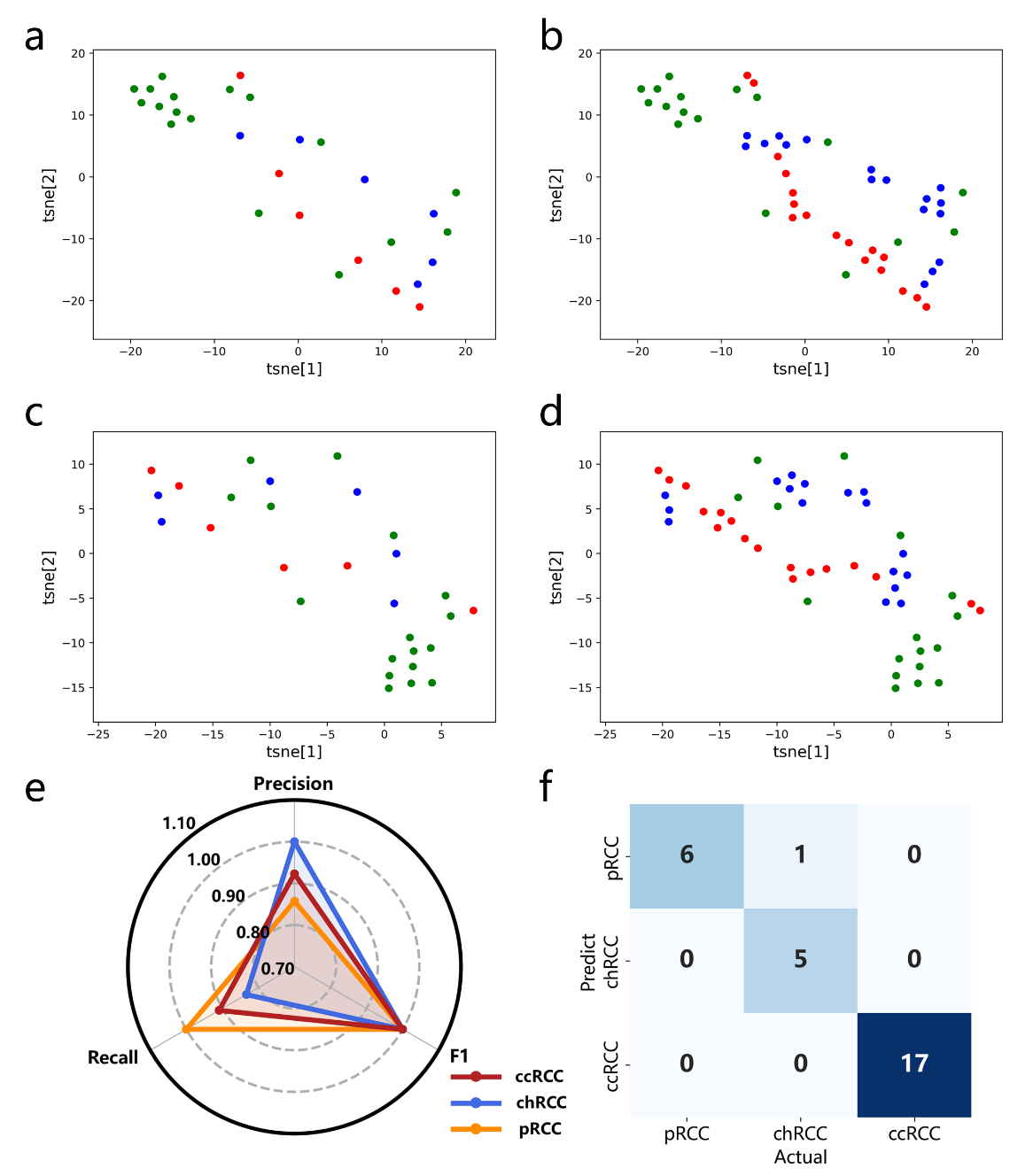


**Figure S3**

Multi-classification model. (a-b) The t-SNE of 3 RCC subtypes (perplexity = 5), including pRCC (red dots), chRCC (blue dots), and ccRCC (green dots): raw data (a) and after smote (b). (c-d) The t-SNE of 3 RCC subtypes (perplexity = 6), including pRCC (red dots), chRCC (blue dots), and ccRCC (green dots): raw data (a) and after smote (b). (e) Model evaluation of 3 subtypes of RCC based on the final optimized model, including Precision, Recall, and F1. (f) Confusion matrix for the classification results of the proposed algorithm on the discovery dataset (6 pRCC, 6 chRCC, and 17 ccRCC).

**Figure S4**


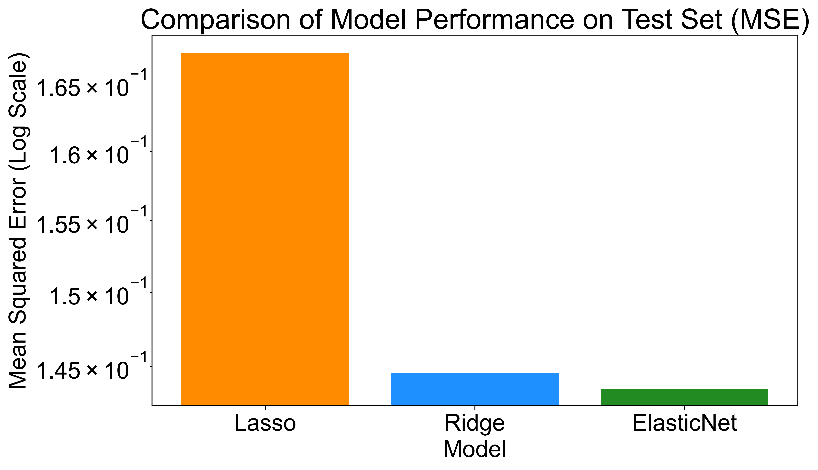


**Figure S4**

Comparison of model performance by mean squared error (MSE).

**Figure S5**


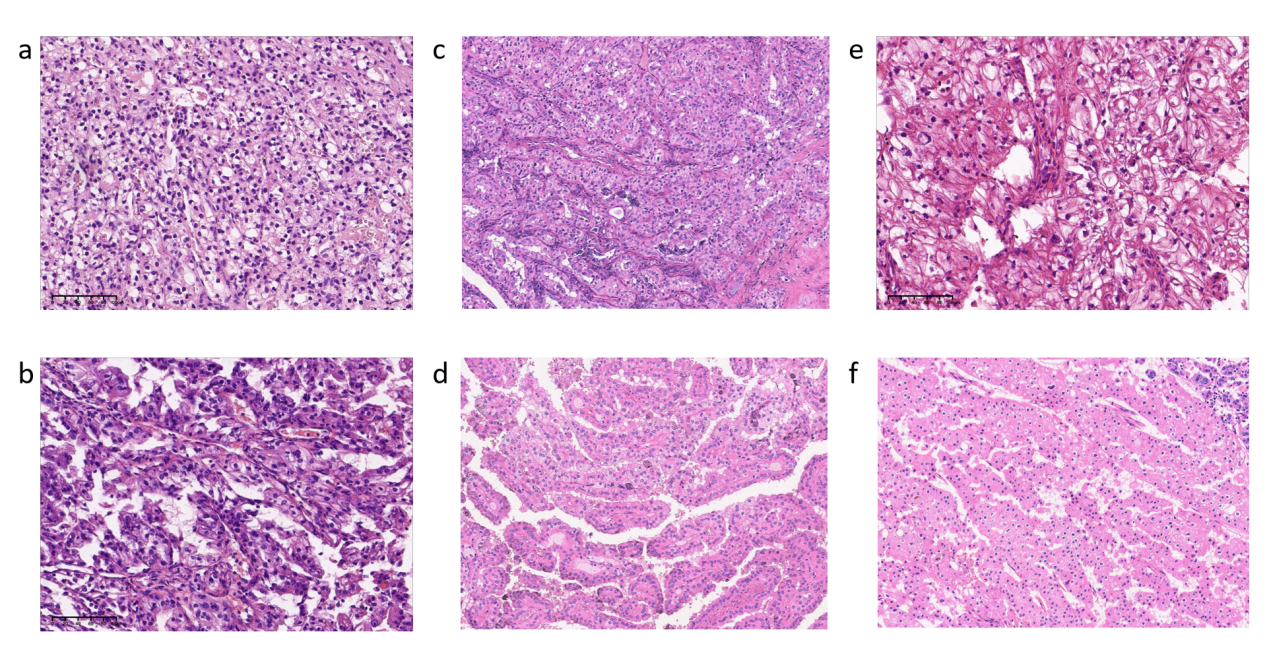


**Figure S5**

Pathology tissue slides of RCC patients at different stages. The pathology tissue slides of RCC patients showed different stages of (a) Stage I of ccRCC, (b) Stage III of ccRCC, (c) Stage I of pRCC, (d) Stage III of pRCC, (e) Stage of chRCC, and (f) Stage IV of chRCC.

**Figure S6**


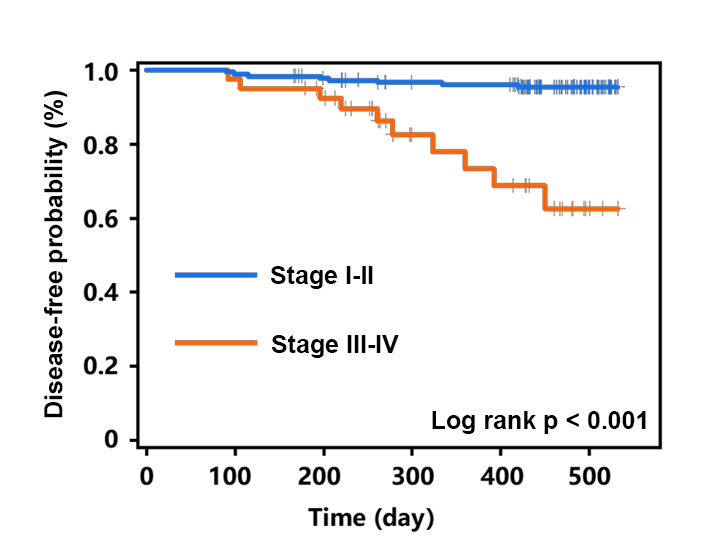


**Figure S6**

Disease-free analysis for ccRCC patients by TNM stage system.

**Figure S7**

**
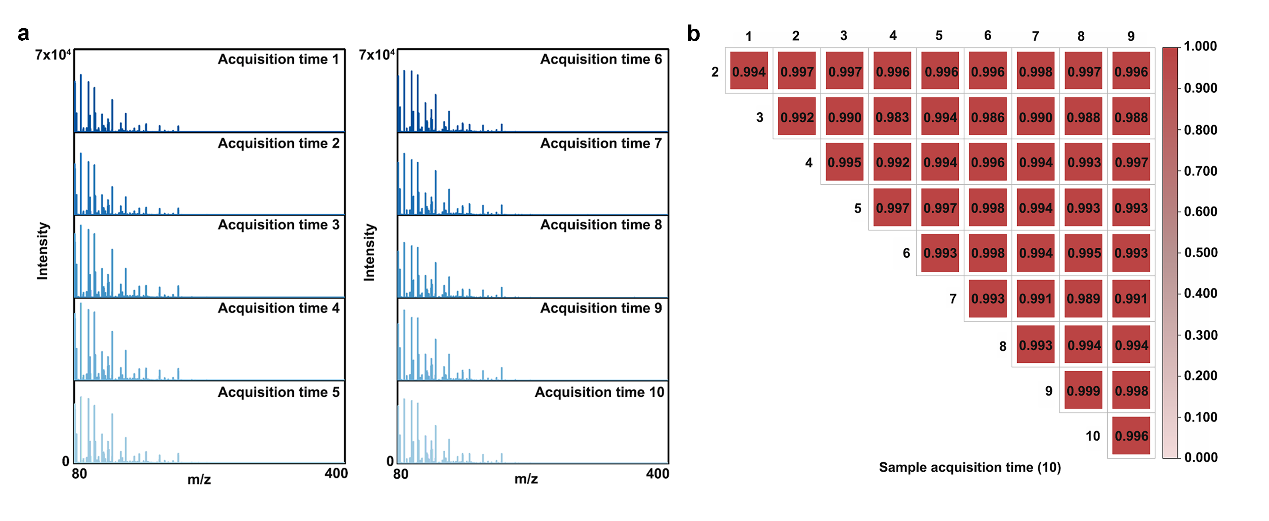
**

**Figure S****7**

Sample reproducibility. (a) 10 mass spectra from the same sample at different time points; (b) Intraclass correlation coefficients between 10 mass spectra.

**Figure S8**

**
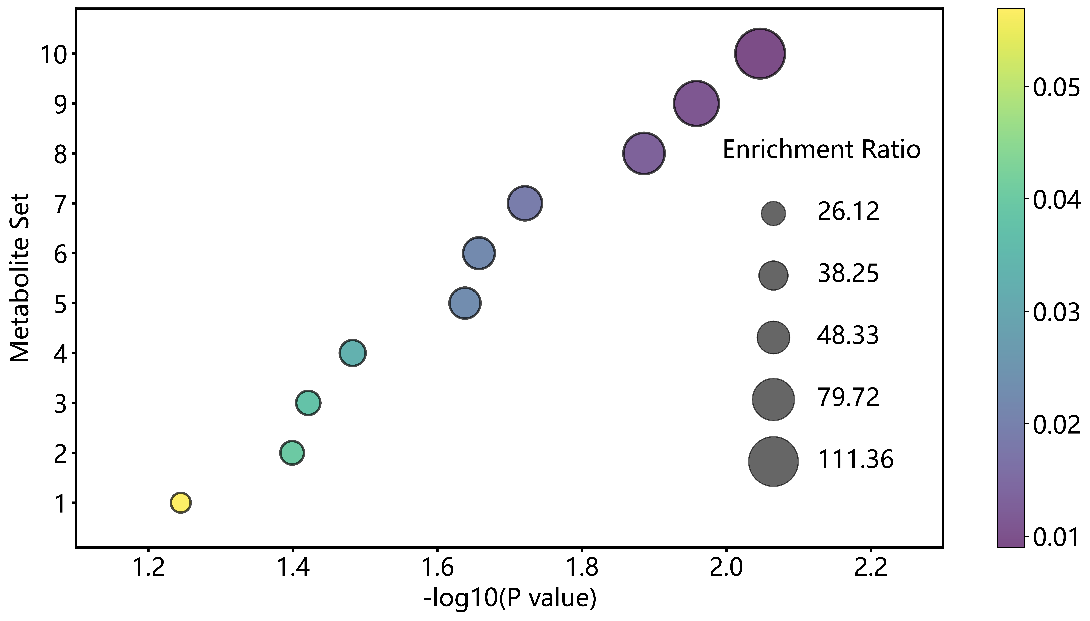
**

**Figure S8**

Potential pathways related to the selected metabolites for RCC subtype diagnosis. The color and size of each circle corresponded to the p value and enrichment ratio. Ten pathways were screened out, including 1) Warburg Effect, 2) Sphingolipid Metabolism, 3) Galactose Metabolism, 4) Gluconeogenesis, 5) Glycolysis, 6) Transfer of Acetyl Groups into Mitochondria, 7) Lactose Synthesis, 8) Glucose-Alanine Cycle, 9) Trehalose Degradation, and 10) Lactose Degradation.

**Figure S9**


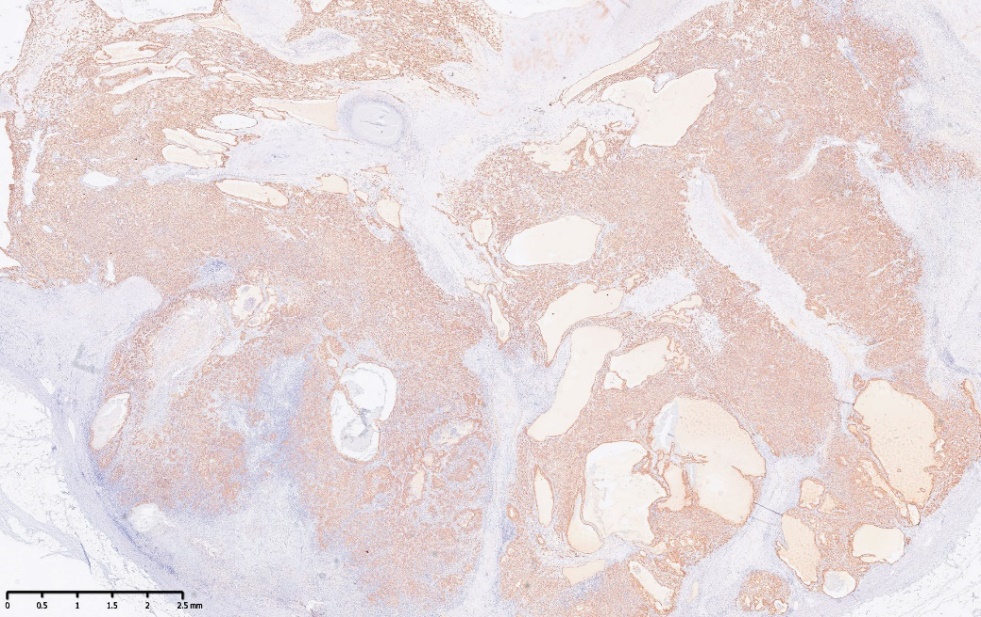


**Figure S9**

CA-IX antibody result of one ccRCC patient.

| **Table S1. Clinical characteristics of the enrolled Renal cell carcinoma group (n = 565).** | | | |
| --- | --- | --- | --- |
| **Clinical indexes** | | **Number** | **%** |
| Sex |  |  |  |
|  | Female | 229 | 40.5 |
|  | Male | 336 | 59.5 |
| Age (years) | |  |  |
|  | <60 | 337 | 59.6 |
|  | ≥60 | 228 | 40.4 |
| BMI |  |  |  |
|  | <24 | 276 | 48.9 |
|  | ≥24 | 289 | 51.1 |
| Pathological classification | |  |  |
|  | ccRCC | 243 | 43.0 |
|  | pRCC | 117 | 20.7 |
|  | chRCC | 120 | 21.2 |
|  | AML | 41 | 7.3 |
|  | other types | 44 | 7.8 |
| Pathological stage (except AML) | |  |  |
|  | I | 352 | 67 |
|  | II | 104 | 20 |
|  | III-IV | 68 | 13 |
| ISUP grade | |  |  |
|  | I | 86 | 24 |
|  | II | 182 | 51 |
|  | III-IV | 92 | 25 |

| **Table S2. Clinical characteristics of other types (n = 44).** | | | | | |
| --- | --- | --- | --- | --- | --- |
| ID | Sex | Age | BMI | TNM | Type |
| O1 | male | 60 | 29.05 | T1aN0M0 | oncocytoma |
| O2 | female | 31 | 19.33 | T1aN0M0 | neuroendocrine tumor |
| O3 | male | 59 | 25.06 | T1aN0M0 | renal cyst |
| O4 | male | 63 | 22.99 | T1bN1M1 | invasive high-grade urothelial carcinoma |
| O5 | female | 34 | 23.34 | T1bN0M0 | Xp11.2/TFE3 translocation renal cell carcinoma |
| O6 | male | 28 | 18.81 | / | VHL |
| O7 | male | 45 | 24.97 | T1bN0M0 | multilocular cystic renal neoplasm of low malignant potential |
| O8 | female | 55 | 28.04 | T3aN1M1 | Mucinous tubular and spindle cell renal cell carcinoma |
| O9 | male | 69 | 23.39 | T1aN0M0 | biphasic papillary renal cell carcinoma |
| O10 | male | 69 | 25.40 | T1bN0M0 | clear cell papillary renal cell carcinoma |
| O11 | male | 67 | 23.03 | T1bN0M0 | unclassified renal cell carcinoma |
| O12 | female | 46 | 23.14 | / | epithelioid angiomyolipoma |
| O13 | female | 29 | 22.31 | / | juxtaglomerular cell tumor |
| O14 | male | 62 | 25.83 | / | adrenal sebaceous adenoma |
| O15 | female | 46 | 22.64 | T1aN0M0 | multilocular cystic renal neoplasm of low malignant potential |
| O16 | male | 32 | 27.04 | / | renal cyst |
| O17 | female | 55 | 24.00 | T1aN0M0 | oncocytoma |
| O18 | male | 47 | 33.26 | T1bN0M0 | oncocytoma |
| O19 | male | 49 | 20.76 | T1aN0M0 | oncocytoma |
| O20 | male | 58 | 25.31 | T1aN0M0 | renal cyst |
| O21 | male | 18 | 24.11 | T1bN0M0 | Xp11.2/TFE3 translocation renal cell carcinoma |
| O22 | male | 24 | 27.41 | T1bN1M0 | chronic granulomatous inflammation |
| O23 | female | 43 | 21.45 | T1bN0M0 | epithelioid angiomyolipoma |
| O24 | female | 61 | 21.26 | T1aN0M0 | papillary renal cell carcinoma with eosinophilic cytoplasmatic |
| O25 | male | 48 | 22.59 | T1bN0M0 | clear cell papillary renal cell carcinoma |
| O26 | male | 38 | 23.53 | T1aN0M0 | Xp11.2/TFE3 translocation renal cell carcinoma |
| O27 | female | 57 | 24.52 | T1aN0M0 | unclassified renal cell carcinoma with eosinophilic cytoplasmatic |
| O28 | male | 30 | 20.28 | T2aN1M0 | unclassified renal cell carcinoma, grade II-III |
| O29 | male | 72 | 26.35 | T1bN0M0 | unclassified renal cell carcinoma, grade II |
| O30 | female | 46 | 28.12 | / | nephridial tissue |
| O31 | female | 50 | 22.58 | T1aN0M0 | oncocytoma |
| O32 | female | 66 | 24.03 | T1aN0M0 | clear cell papillary renal cell carcinoma |
| O33 | female | 48 | 22.66 | T1aN0M0 | clear cell papillary renal cell carcinoma |
| O34 | male | 52 | 23.53 | T1bN0M0 | multilocular cystic renal neoplasm of low malignant potential |
| O35 | female | 40 | 26.99 | / | multilocular renal cyst |
| O36 | female | 43 | 25.71 | T3aN0M0 | Xp11.2/TFE3 translocation renal cell carcinoma |
| O37 | female | 67 | 26.03 | T1aN0M0 | unclassified renal cell carcinoma |
| O38 | male | 77 | 24.22 | T1aN0M0 | clear cell papillary renal cell carcinoma |
| O39 | male | 71 | 20.02 | T1aN0M0 | oncocytoma |
| O40 | male | 57 | 20.76 | T1aN0M0 | unclassified renal cell carcinoma |
| O41 | female | 33 | 32.02 | / | epithelioid angiomyolipoma |
| O42 | male | 47 | 25.69 | / | epithelioid angiomyolipoma |
| O43 | male | 43 | 24.34 | / | epithelioid angiomyolipoma |
| O44 | male | 58 | 26.70 | / | proliferative lesions of lymph tissue |

| **Table S3. Clinical characteristics of the healthy control group.** | | | |
| --- | --- | --- | --- |
| **Clinical indexes** | | **Number** | **%** |
| Sex |  |  |  |
|  | Female | 94 | 47 |
|  | Male | 106 | 53 |
| Age(years) | |  |  |
|  | <60 | 168 | 84 |
|  | ≥60 | 32 | 16 |
| BMI |  |  |  |
|  | <24 | 123 | 61.5 |
|  | ≥24 | 87 | 43.5 |

| **Table S4. Variables of RCC subtypes.**  **Variables in the Equation (ccRCC)** | | | | | | | |
| --- | --- | --- | --- | --- | --- | --- | --- |
| Step 1^a^ | Covariates | B | S.E. | Wald | df | Sig. | Exp(B) |
|  | Gender | -0.255 | 0.213 | 1.433 | 1 | 0.231 | 0.775 |
|  | BMI | 0.72 | 0.33 | 4.822 | 1 | 0.028 | 1.074 |
|  | Constant | -1.053 | 0.819 | 1.653 | 1 | 0.199 | 0.349 |
| Variable(s) entered on step 1: Gender, BMI. | | | | | | | |

| **Variables in the Equation (pRCC)** | | | | | | | |
| --- | --- | --- | --- | --- | --- | --- | --- |
| Step 1^b^ | Covariates | B | S.E. | Wald | df | Sig. | Exp(B) |
|  | Gender | -0.448 | 0.284 | 2.488 | 1 | 0.115 | 0.639 |
|  | BMI | 0.053 | 0.042 | 1.594 | 1 | 0.207 | 1.054 |
|  | Constant | -1.526 | 1.058 | 2.080 | 1 | 0.149 | 0.217 |
| Variable(s) entered on step 1: Gender, BMI. | | | | | | | |

| **Variables in the Equation (chRCC)** | | | | | | | |
| --- | --- | --- | --- | --- | --- | --- | --- |
| Step 1^c^ | Covariates | B | S.E. | Wald | df | Sig. | Exp(B) |
|  | Gender | 0.964 | 0.294 | 10.785 | 1 | 0.001 | 2.622 |
|  | BMI | 0.149 | 0.043 | 11.881 | 1 | 0.001 | 1.161 |
|  | Constant | -4.605 | 1.133 | 16.527 | 1 | 0.000 | 0.010 |
| Variable(s) entered on step 1: Gender, BMI. | | | | | | | |

**Table S5. Best model and optimal parameter for different tasks.**

| Tasks | Subjects | Best model | Optimal parameter |
| --- | --- | --- | --- |
| Tumor  Screening | Tumors vs. HC | Ridge | alpha=1.0; tol=0.0001 |
| Tumor Discrimination | RCC vs. AML | ExtraTrees | criterion='gini';  max_features='sqrt' |
| Subtype Classification | ccRCC vs. pRCC vs. chRCC | RandomForest | n_estimators=100;  class_weight='balanced' |
| Early  Diagnosis | ccRCC vs. HC | LGBM | colsample_bytree=1.0;  n_estimators=100; num_leaves=31 |
|  | pRCC vs. HC | LGBM | colsample_bytree=1.0; n_estimators=100; num_leaves=31 |
|  | chRCC vs. HC | Ridge | alpha=1.0; tol=0.0001 |

| Table S6. Summary of performances of models for early-stage ccRCC diagnosis. | | | | | | | | |
| --- | --- | --- | --- | --- | --- | --- | --- | --- |
| Model | **Accuracy** | **AUC** | **Recall** | **Precision** | **F1** | **Kappa** | **MCC** | **TT (Sec)** |
| LGBM | 0.955 | 0.985 | 0.953 | 0.965 | 0.959 | 0.909 | 0.910 | 0.148 |
| ERT | 0.942 | 0.986 | 0.940 | 0.955 | 0.946 | 0.882 | 0.886 | 0.158 |
| RF | 0.929 | 0.990 | 0.929 | 0.946 | 0.936 | 0.857 | 0.862 | 0.160 |
| AB | 0.923 | 0.984 | 0.940 | 0.929 | 0.930 | 0.842 | 0.852 | 0.173 |
| GBC | 0.910 | 0.982 | 0.917 | 0.921 | 0.918 | 0.817 | 0.819 | 0.267 |
| DT | 0.844 | 0.839 | 0.907 | 0.838 | 0.863 | 0.682 | 0.701 | 0.138 |
| KNN | 0.837 | 0.922 | 0.867 | 0.843 | 0.847 | 0.671 | 0.685 | 0.285 |
| LR | 0.806 | 0.906 | 0.836 | 0.820 | 0.610 | 0.621 | 0.621 | 0.391 |
| LDA | 0.701 | 0.736 | 0.689 | 0.745 | 0.711 | 0.401 | 0.408 | 0.141 |
| NB | 0.663 | 0.808 | 0.538 | 0.765 | 0.617 | 0.345 | 0.363 | 0.136 |

Abbreviations: LGBM, Light Gradient Boosting Machine; ERT, Extremely Randomized Trees; RF, Random Forest; AB, AdaBoost; GBC, Gradient Boosting Classifier; DT, Decision Tree; KNN, k-Nearest Neighbors; LR, Logistic Regression; LDA, Linear Discriminant Analysis; NB, Naive Bayes.

| Table S7. Summary of performances of models for early-stage pRCC diagnosis. | | | | | | | | |
| --- | --- | --- | --- | --- | --- | --- | --- | --- |
| Model | **Accuracy** | **AUC** | **Recall** | **Precision** | **F1** | **Kappa** | **MCC** | **TT (Sec)** |
| LGBM | 0.874 | 0.940 | 0.923 | 0.850 | 0.872 | 0.746 | 0.769 | 0.145 |
| AB | 0.849 | 0.904 | 0.873 | 0.846 | 0.846 | 0.695 | 0.714 | 0.175 |
| GBC | 0.832 | 0.914 | 0.840 | 0.834 | 0.826 | 0.662 | 0.676 | 0.319 |
| LR | 0.774 | 0.832 | 0.733 | 0.783 | 0.745 | 0.542 | 0.554 | 0.362 |
| RF | 0.766 | 0.804 | 0.803 | 0.771 | 0.773 | 0.531 | 0.554 | 0.160 |
| LDA | 0.744 | 0.791 | 0.723 | 0.732 | 0.715 | 0.484 | 0.492 | 0.135 |
| ERT | 0.732 | 0.784 | 0.723 | 0.757 | 0.716 | 0.457 | 0.477 | 0.151 |
| KNN | 0.705 | 0.716 | 0.640 | 0.719 | 0.662 | 0.400 | 0.411 | 0.276 |
| DT | 0.619 | 0.612 | 0.493 | 0.625 | 0.527 | 0.226 | 0.238 | 0.131 |
| NB | 0.596 | 0.653 | 0.430 | 0.568 | 0.473 | 0.179 | 0.179 | 0.128 |

Abbreviations: LGBM, Light Gradient Boosting Machine; AB, Adaptive Boosting; GBC, Gradient Boosting Classifier; LR, Logistic Regression; RF, Random Forest; LDA, Linear Discriminant Analysis; ERT, Extra Randomized Trees; KNN, k-Nearest Neighbors; DT, Decision Tree; NB, Naive Bayes.

| Table S8. Summary of performances of models for early-stage chRCC diagnosis. | | | | | | | | |
| --- | --- | --- | --- | --- | --- | --- | --- | --- |
| Model | **Accuracy** | **AUC** | **Recall** | **Precision** | **F1** | **Kappa** | **MCC** | **TT (Sec)** |
| Ridge | 0.906 | 0.931 | 0.900 | 0.915 | 0.903 | 0.812 | 0.820 | 0.126 |
| GBC | 0.899 | 0.912 | 0.843 | 0.957 | 0.891 | 0.798 | 0.810 | 0.132 |
| AB | 0.871 | 0.916 | 0.814 | 0.926 | 0.861 | 0.742 | 0.754 | 0.132 |
| LR | 0.856 | 0.946 | 0.798 | 0.907 | 0.844 | 0.712 | 0.723 | 0.365 |
| LGBM | 0.856 | 0.904 | 0.829 | 0.877 | 0.849 | 0.712 | 0.717 | 0.132 |
| RF | 0.828 | 0.850 | 0.800 | 0.917 | 0.810 | 0.685 | 0.734 | 0.119 |
| LDA | 0.819 | 0.879 | 0.795 | 0.840 | 0.811 | 0.636 | 0.645 | 0.130 |
| DT | 0.818 | 0.817 | 0.810 | 0.845 | 0.813 | 0.634 | 0.656 | 0.132 |
| ERT | 0.813 | 0.895 | 0.769 | 0.855 | 0.804 | 0.626 | 0.637 | 0.145 |
| NB | 0.725 | 0.787 | 0.583 | 0.838 | 0.667 | 0.453 | 0.488 | 0.130 |
| KNN | 0.675 | 0.694 | 0.538 | 0.745 | 0.609 | 0.351 | 0.372 | 0.269 |
| QDA | 0.550 | 0.552 | 0.538 | 0.559 | 0.541 | 0.105 | 0.107 | 0.129 |

Abbreviations: Ridge, Ridge Regression; GBC, Gradient Boosting Classifier; AB, Adaptive Boosting; LR, Logistic Regression; LGBM, Light Gradient Boosting Machine; RF, Random Forest; LDA, Linear Discriminant Analysis; DT, Decision Tree; ERT, Extra Randomized Trees; NB, Naive Bayes; KNN, k-Nearest Neighbors; QDA, Quadratic Discriminant Analysis.

**Table S9. Serum biomarkers were validated using Fourier-transform ion-cyclotron resonance mass spectrometry (FT-ICR MS).**

| **Biomarkers** | **Metabolites** | **Molecular formula** | **Adducts** | **Error (ppm)** | **Related subtype** |
| --- | --- | --- | --- | --- | --- |
| S113.6 | 6-Methyltetrahydropterin | C_7_H_11_N_5_O | M + 2Na | 5.89 | ccRCC |
| S151.1 | L-Cyclo(alanyl glycyl) | C_5_H_8_N_2_O_2_ | M + Na | 2.09 | ccRCC |
| S179.1 | D-Gluconic acid | C_6_H_10_O_6_ | M + H | 1.55 | ccRCC |
| S135.0 | D-Malic acid | C_4_H_6_O_5_ | M + H | 1.41 | chRCC & pRCC |
| S203.0 & S203.1 | D-Glucose | C_6_H_12_O_6_ | M + Na | 1.05 | chRCC & pRCC |
| S140.8 | Mesoxalic acid | C_3_H_2_O_5_ | M + Na | 4.46 | chRCC |

**Table S10. Urine biomarkers were validated using FT-ICR MS.**

| **Biomarkers** | **Metabolites** | **Molecular formula** | **Adducts** | **Error (ppm)** | **Related subtype** |
| --- | --- | --- | --- | --- | --- |
| U100.1 | 2-Piperidinone | C_5_H_9_NO | M+H | 3.64 | ccRCC |
| U143.0 & U143.1 | Sulfobetaine | C_4_H_8_O_2_S | M+Na | 1.97 | pRCC & ccRCC |
| U186.8 | Deoxycholylvaline | C_29_H_49_NO_5_ | M+3Na | 1.56 | ccRCC |
| U242.9 | Inositol cyclic phosphate | C_6_H_11_O_8_P | M+H | 1.65 | ccRCC |
| U103.9 | N-Formylglycine | C_6_H_12_O_6_ | M+H | 6.94 | pRCC |
| U152.0 | Pipecolic acid | C_6_H_11_NO_2_ | M+Na | 1.99 | pRCC & chRCC |
| U256.0 | Aminobutane bisphosphonate | C_4_H_13_NO_6_P_2_ | M+Na | 0.19 | pRCC |
| U119.9 | Phosphoramidic acid | H_4_NO_3_P | M+Na | 3.97 | chRCC |
| U156.8 | Glycohyocholate | C_26_H_42_NO_6_ | M+3H | 3.9 | chRCC |
